# Generalized Statistical Isotherm for Modeling Adsorption Equilibria in Stimuli-Responsive Framework Materials

**DOI:** 10.1021/acs.langmuir.6c01192

**Published:** 2026-06-17

**Authors:** Hassan Azzan, David Danaci, Camille Petit, Ronny Pini

**Affiliations:** † Department of Chemical Engineering, 4615 Imperial College London, London SW7 2AZ, United Kingdom; ‡ The Sargent Centre for Process Systems Engineering, Imperial College London, London SW7 2AZ, United Kingdom; § I-X Centre for AI in Science, Imperial College London, London W12 0BZ, United Kingdom

## Abstract

Designing large-scale adsorption-based separation units requires a multiscale understanding of material behavior, from molecular interactions to process-level performance. An underpinning challenge is the development of predictive multicomponent adsorption equilibrium models for systems departing from the classic type I isotherm for microporous adsorbents. Adsorbents that undergo adsorption-induced transitions (flexible adsorbents) belong to this category and are a class of framework materials that exhibit unique features such as sigmoidal equilibrium isotherms and intrinsic thermal management capabilities. Despite their promise for practical applications, flexible adsorbents remain underexplored at the process scale due to the lack of a suitable equilibrium isotherm model that mechanistically captures their adsorption-induced structural transitions. Here, we present a simplified statistical isotherm model for transition materials (SSI-T) as a generalized approach to parametrize—using a combination of sorbate-dependent and -independent physical parameters—the adsorption equilibria in flexible adsorbents that exhibit a broad range of structural and configurational transitions upon adsorption. We have validated the model using unary and binary equilibrium data for gate-opening, breathing, and configurational transitions in multiple adsorbent–adsorbate systems. Our formulation represents the first continuous, differentiable, and explicit isotherm model in the literature capable of accurately describing unary adsorption and desorption isotherms in flexible adsorbents, and predicting binary equilibria for multiple types of adsorption-induced transitions without any additional parameters. Because SSI-T is an explicit function of state variables, it can be seamlessly integrated into process-scale simulators, enabling the design and optimization of adsorption-based technologies that use flexible adsorbents.

## Graphical abstract

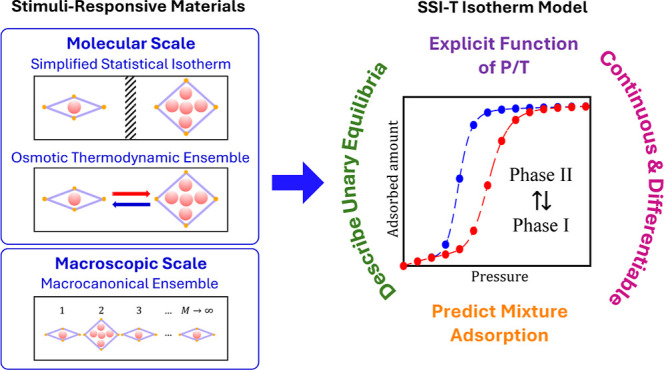

## 1 Introduction

Adsorption-based technology utilizing porous adsorbents holds significant promise in addressing pressing global challenges, including global warming, water scarcity, and energy security. Mature adsorbents such as zeolites have been a mainstay in applications such as hydrogen purification, catalysis, and oxygen generation due to their thermal stability and high selectivity. Similarly, activated carbons are inexpensive to produce and versatile for various applications, including water purification and capturing volatile organic compounds (VOCs). However, the rigidity of these materials, in terms of their pore structure and chemical composition, often results in undesired features such as low selectivity in carbons in general and hydrophilicity in most zeolites, leading to low selectivity under wet conditions.

One of the pivotal developments in materials science and reticular chemistry in recent decades has been the development of functional porous coordination polymers,^1^ such as metal–organic frameworks (MOFs),^2,3^ which have created new avenues for addressing the shortcomings of existing adsorbent classes. Relative to zeolites, porous coordination polymers consist of coordination entities repeating in up to three dimensions,^4^ and can be constructed with a wide array of building blocks under milder conditions. This opens the door to designing more complex modular structures with greater porosity and tunable functionality.^5^ Unsurprisingly, research into design and discovery of these materials accelerated markedly over the past two decades, with over half a million hybrid metal–organic structures documented in the Cambridge Structural Database as of 2023.^6^

Stimuli-responsive materials, also referred to as soft-porous crystals,^7^ form the third generation of porous coordination polymers—as classified by Kitagawa et al.—consist of repeating units that can be triggered to reversibly transition between two or more stable structural phases using external stimuli such as light, magnetic fields, or adsorbed molecules as illustrated in Figure 1. Stimuli-responsive materials that undergo guest-induced phase transitions are of particular interest in adsorption-based applications. In contrast to conventional adsorbents with essentially rigid (or frozen^8^) pore structures and thus fixed thermodynamic and kinetic properties, these materials exhibit adsorption-induced structural or configurational transitions of relatively large magnitudes, as illustrated in Figure 1, often producing sigmoidal or hysteretic adsorption isotherms. For a more in-depth review from the perspective of reticular chemistry and molecular modeling, we refer the reader to recent review articles.^9,10^ These materials, although not always flexible in structure, are commonly classed collectively as *flexible adsorbents* and will be referred to as such in this article.

**Figure 1:**
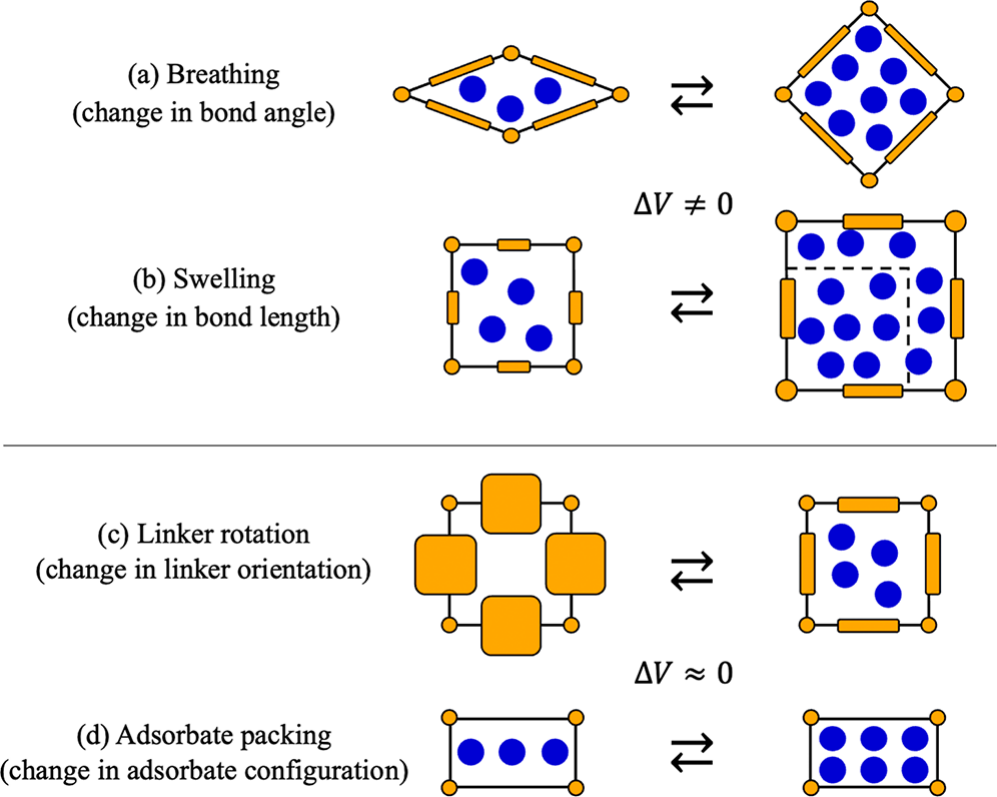
Schematic illustration of some examples of types of adsorption-induced transition separated into those that result in a change in the void volume of the adsorbent available to guest molecules (top two) and those that do not result in a volume change (bottom two). (a) Breathing transitions are a specific case of structural transitions characterized by a change in bond angle between linkers, resulting in a volume change, and the open phase is more stable under vacuum. (b) Swelling transitions are characterized by a change in bond length between metal nodes, resulting in a volume change. (c) Linker rotation or gating transitions are characterized by low or no adsorption in the closed phase and higher adsorption in the open phase, without a volume change of the framework, e.g. gating transitions. (d) Some transitions are characterized by how the adsorbate molecules are packed or configured within the framework and may result in sigmoidal isotherms—these may also be referred to as configurational transitions.

From an application perspective, the transition properties of flexible adsorbents carry significant promise in addressing pressing energy and environmental applications such as gas storage, atmospheric water harvesting, and greenhouse gas capture. From a basic thermodynamic assessment, flexible adsorbents that exhibit significantly different adsorption properties between their structural phases and also exhibit transitions within realistic conditions (e.g., transition below the feed partial pressure for the strongly adsorbing component in a pressure-swing adsorption process) can potentially allow flexible adsorbents to outperform conventional adsorbents in terms of productivity and regenerability.^11–15^ From a storage perspective, for instance, flexible adsorbents that exhibit hysteresis can be loaded (with e.g. methane or hydrogen) at high pressure (along the adsorption branch), and depressurized for storage at a lower pressure (on the desorption branch) without losing stored capacity, potentially reducing the safety hazards of storing flammable and explosive gases and the capital expenditure required for high-pressure equipment and their operation. From a separation application, e.g. in a pressure swing adsorption process to separate a mixture of two or more species, the isotherm inflection point—if appropriately located—can provide a sufficient working capacity for the target species without requiring vacuum levels as low as those needed for *rigid* adsorbents that exhibit a concave (type I) adsorption isotherm with respect to pressure.

While the above discussion is valuable in identifying potential adsorption-based applications for flexible adsorbents, a more rigorous engineering approach focusing beyond basic thermodynamic properties is required to quantify their feasibility for large-scale deployment (i.e., by accounting for properties such as adsorption kinetics, process design, and operating conditions). The first step for designing an adsorption-based process for a given adsorbate–adsorbent system is to predict its performance using numerical process simulators.^16–20^ Common to all of them is the need to be parametrized with both theoretical and empirical parameters that describe various adsorbent, adsorbate, and bed properties. In this endeavor, one, if not the most important step, is the parametrization of the equilibrium adsorption behavior of the relevant species present using an ideal isotherm model.^21,22^ We can define an isotherm model as *ideal* for use within a process modeling framework by ensuring it meets the following criteria.

1. **Describe unary (single adsorbate) equilibrium**: The model should be able to reproduce the unary adsorption equilibrium data (for adsorption and desorption)-obtained experimentally or through molecular-scale modeling and simulation tools-for all the species present in the process being modeled.
2. **Predict mixture adsorption**: The model should be able to predict the adsorption of the individual species at equilibrium, subject to competition/cooperation between species for the available capacity within the adsorbent, without any additional parameters to the unary descriptors.
3. **Continuous and differentiable**: Numerical methods used to obtain continuous solutions to process model equations require the equilibrium adsorbed amount to also be modeled in a continuous and differentiable manner with respect to state variables (e.g., composition, pressure, temperature). For hysteretic systems, the isotherm model should also be able to model within the phase coexistence boundary.
4. **Explicit function of state variables**: The model should be an explicit function of the state variables pressure, temperature, and composition for computational efficiency.

The past two decades have seen the development of useful theoretical frameworks that have advanced the modeling of adsorption in flexible adsorbents.^9^ The osmotic thermodynamic ensemble^23^ provides a generalized expression for the thermodynamic potential of a host–guest system defined as the osmotic potential Ω^os^ in terms of the free energy of host *F* ^host^, the external pressure *p*, and the adsorption isotherm of the guest species *N* ^ads^ of molar volume *V*_m_ within a unit cell volume of *v*_uc_, as given by eq 1.

1 $$\Omega^{os}\left(p,T,v_{uc}\right)=-\int_{0}^{p}N^{ads}\left(p,T\right)V_{m}\left(p,T\right)dP+pv_{uc}+F^{host}\left(p,T,v_{uc}\right)$$

For a given host–guest system, a guest-induced transition between two distinct phases will be observed if there exists a state where the osmotic potential computed for each phase is equal. If both phases are assumed to be rigid, i.e., *v*_uc_ is constant and hence, *F* ^host^ is independent of the adsorbed amount, the osmotic ensemble predicts a first-order discontinuous transition at the conditions where Ω_I_^os^ = Ω_II_^os^. For some systems, the threshold adsorbed phase potential required to induce a transition varies depending on the direction of the transition, resulting in hysteresis in the measured isotherms. Neimark et al.^24^ proposed a stress-based model to describe this behavior in breathing flexible adsorbents, which applies to other similar systems. The osmotic ensemble is readily extended to mixed-gas adsorption through the osmotic framework adsorbed solution theory (OFAST), which involves computing the thermodynamic potential of the adsorbed molecules (first term in eq 1) for the total number of adsorbed molecules of the mixture at equilibrium instead of the single adsorbate.^25^ Several analytical models have been proposed in the literature that adopt the osmotic ensemble and extend it to describe continuous phase transitions as observed in macroscopic systems.

Hiraide et al. applied the grand canonical ensemble to the osmotic ensemble to propose a general form of an isotherm model (structural transition type adsorption, STA isotherm) and modeled continuous guest-induced transitions between two phases described by either Langmuir or Sips isotherms.^26^ While this model can accurately describe unary adsorption in flexible adsorbents, the use of models such as Langmuir and Sips (which assume a fixed number of adsorption sites in the host) results in physical inconsistencies in fitted parameters, such as different values for Δ*F* ^host^ depending on the adsorbate and the choice of model used to describe the individual phases. Thus, the STA model, as proposed, cannot be extended to model mixture adsorption in flexible adsorbents. Similar inconsistencies have been reported for other empirical and semiempirical models for such systems.^27,28^ These models, although satisfying three of the four required features above, cannot predict mixture adsorption based on unary parametrization.

Dunne and Manos^29^ presented an analytical lattice model that can describe continuous adsorption-induced breathing transitions. Here, a continuous transition with state variables is obtained by modeling interactions between neighboring unit cells with the addition of empirical interaction parameters, and the formulation reduces to the osmotic ensemble in its simplest form. Verbraeken and Brandani^8^ proposed a model based on the rigid adsorbent lattice fluid (RALF)^30^ model, consisting of fewer adjustable parameters that can predict binary adsorption in breathing flexible adsorbents by allowing for different adsorbed phase densities of the dissimilar species. However, since the host–guest system is treated as a unit cell in isolation, the implicit solution of the model equation results in a discontinuous transition and requires an empirical function to obtain a continuous isotherm. In addition, both of these approaches require the implicit approach to compute the adsorbed amount, and thus are not ideal for application in process simulation and design.

In this work, we propose an explicit isotherm model to describe continuous guest-induced transitions between two essentially rigid phases of a flexible adsorbent. To our knowledge, this is the first mechanistic isotherm model for flexible adsorbents that meets the criteria described above for use within an adsorption process modeling workflow. The model accounts for hysteresis and can predict binary adsorption of dissimilar sorbate molecules using the parametrization of unary adsorption and desorption isotherms. The resulting simplified statistical isotherm model for transition materials (SSI-T) consists of five sorbate-dependent parameters to describe the adsorption in the two phases, and five sorbate–invariant parameters to describe the structure and free energy of the host framework. The adsorption in each phase is characterized by the simplified statistical isotherm (SSI) proposed initially by Ruthven for zeolites.^31^ The condition for a phase transition between two rigid phases described by the SSI model is given by the osmotic ensemble, which is then used to model a continuous isotherm through the approximation of phase-coexistence at the macroscopic limit to yield the proposed model for a reversible and hysteretic systems. The SSI-T model is then extended to predict mixture adsorption without any additional parametrization. We validate the unary parametrization of the model on different flexible adsorbents reported in the literature that undergo various forms of structural and configurational transitions, and present the prediction of binary adsorption equilibria on three systems of varying complexity. We conclude with a comparison of the SSI-T model with a previously reported formulation in the literature and a discussion on the interpretation of the model parameters based on their physical interpretation, including potential implications for the synthesis and formulation of flexible adsorbents for adsorption-based processes.

## 2 Methods

In this section, we present the details of the methodology used in the derivation of the SSI-T model, its features, and its extension to mixed adsorbate systems. We end the section with a c description of the methodology used to estimate the model parameters by fitting the model to unary adsorption/desorption equilibrium data.

### 2.1 Adsorption in a *Frozen* Framework: Simplified Statistical Isotherm

In this section, we introduce and summarize the key features of the simplified statistical isotherm (SSI) model that is used here to model the distinct phases of a flexible adsorbent as essentially *frozen solids* (i.e., solid with a fixed void volume). The complete derivation of the model, as presented by Ruthven^31,32^ for the adsorption of single and binary adsorbates in rigid zeolites, is given in the Supporting Information.

Under standard grand-canonical assumptions for the adsorption of ideal identical molecules in *M* non-interacting cavities, eq 2 defines the canonical partition function $Q^{SSI}$ of the SSI as a function of pressure *p* and temperature *T*.

2 $$Q^{SSI}=Z^{M}={\left[\sum_{i=0}^{\omega }Z\left(i\right){\left(\frac{p}{kT}\right)}^{i}\right]}^{M}$$

here, Z is the canonical partition function for a single cavity, and *Z*(*i*) is the configurational partition function for *i* adsorbed molecules in a cavity given by

3 $$Z\left(i\right)=\frac{{\left(K\left(T\right)kT\right)}^{i}}{i!}{\left(\frac{1-ib/v}{1-b/v}\right)}^{i}$$

where *k* is the Boltzmann constant, *b* is the effective molecular volume of adsorbed molecules considered as hard spheres, *v* is the void volume of the cavity, ω gives the maximum adsorption capacity for the molecules in the cavity (closest integer smaller than the ratio of cavity and effective molecular volumes, ω = ⌊*v*/*b*⌋), and the temperature-dependent Henry’s law constant is given as $K\left(T\right)=K_{0}\;\exp\left[-\Delta u_{ads}/kT\right]$ .

The explicit equation for the ensemble average adsorbed amount of a single adsorbate for a single cavity ${\left\langle N\right\rangle }^{SSI}$ is obtained from eq 4 as a function of *p* and *T*.

4 $${\left\langle N\right\rangle }^{SSI}\left(p,T\right)=\frac{p}{Z}\frac{\partial Z}{\partial p}=\frac{K\left(T\right)p+\sum_{i=2}^{\omega }\frac{{\left(K\left(T\right)p\right)}^{i}}{\left(i-1\right)!}{\left(\frac{1-ib/v}{1-b/v}\right)}^{i}}{1+K\left(T\right)p+\sum_{i=2}^{\omega }\frac{{\left(K\left(T\right)p\right)}^{i}}{i!}{\left(\frac{1-ib/v}{1-b/v}\right)}^{i}}$$

The SSI describes the ensemble average number of adsorbed molecules per cavity [molec. cavity^–1^] and needs to be converted to a mass basis ${\left\langle N\right\rangle }^{SSI,m}$ [mol kg^–1^] to model experimental data. We carry out this conversion by assuming that the average volume of a single cavity is *v* [m^3^ cavity^–1^] and the sum of the total volume of these cavities per unit mass is equal to the micropore volume *v*_mic_ [m^3^ kg^–1^], as given by eq 5.

5 $${\left\langle N\right\rangle }^{SSI,m}=\frac{{\left\langle N\right\rangle }^{SSI}}{N_{A}}\frac{v_{mic}}{v}$$

The extension of this model to predict binary and ternary adsorption equilibria has also been reported.^32–35^ For a system consisting of two adsorbing species α and β, the canonical partition function for a single cavity $Z_{\alpha \beta }$ as a function of the partial pressures *p*_α_ and *p*_β_, and *T* is given by

6 $$Z_{\alpha \beta }=1+K_{\alpha }\left(T\right)p_{\alpha }+K_{\beta }\left(T\right)p_{\beta }+\sum_{j}\sum_{i}\frac{{\left(K_{\alpha }\left(T\right)p_{\alpha }\right)}^{i}{\left(K_{\beta }\left(T\right)p_{\beta }\right)}^{j}}{i!j!}\frac{{\left(1-ib_{\alpha }/v-jb_{\beta }/v\right)}^{i}}{{\left(1-b_{\alpha }/v\right)}^{i}{\left(1-b_{\beta }/v\right)}^{j}},,i+j\geq 2$$

and thus, using single-component parametrizations, the competitive equilibrium adsorbed amount for α in the presence of β is given as follows.

7 $${\left\langle N\right\rangle }_{\alpha }^{SSI}=\frac{p_{\alpha }}{Z_{\alpha \beta }}\frac{\partial Z_{\alpha \beta }}{\partial p_{\alpha }}=\frac{K_{\alpha }\left(T\right)p_{\alpha }+\sum_{j}\sum_{i}\frac{{\left(K_{\alpha }\left(T\right)p_{\alpha }\right)}^{i}{\left(K_{\beta }\left(T\right)p_{\beta }\right)}^{j}}{\left(i-1\right)!j!}\frac{{\left(1-ib_{\alpha }/v-jb_{\beta }/v\right)}^{i}}{{\left(1-b_{\alpha }/v\right)}^{i}{\left(1-b_{\beta }/v\right)}^{j}}}{1+K_{\alpha }\left(T\right)p_{\alpha }+K_{\beta }\left(T\right)p_{\beta }+\sum_{j}\sum_{i}\frac{{\left(K_{\alpha }\left(T\right)p_{\alpha }\right)}^{i}{\left(K_{\beta }\left(T\right)p_{\beta }\right)}^{j}}{i!j!}\frac{{\left(1-ib_{\alpha }/v-jb_{\beta }/v\right)}^{i}}{{\left(1-b_{\alpha }/v\right)}^{i}{\left(1-b_{\beta }/v\right)}^{j}}},,i+j\geq 2$$

### 2.2 Modeling Continuous Adsorption-Induced Structural Transitions

As discussed above, the SSI models adsorption in a frozen solid, and the present work aims to use the above framework to model adsorption in a system where the cavities can exist in exactly two stable phases (frozen states) that may exhibit different adsorption and structural properties. This section provides the derivation of a continuous structural isotherm that describes the adsorption and desorption equilibria subject to guest-induced transitions between the two phases. The model and its features are conceptually illustrated for a hypothetical system that exhibits reversible equilibria in Figure 2 to guide the model description provided.

**Figure 2:**
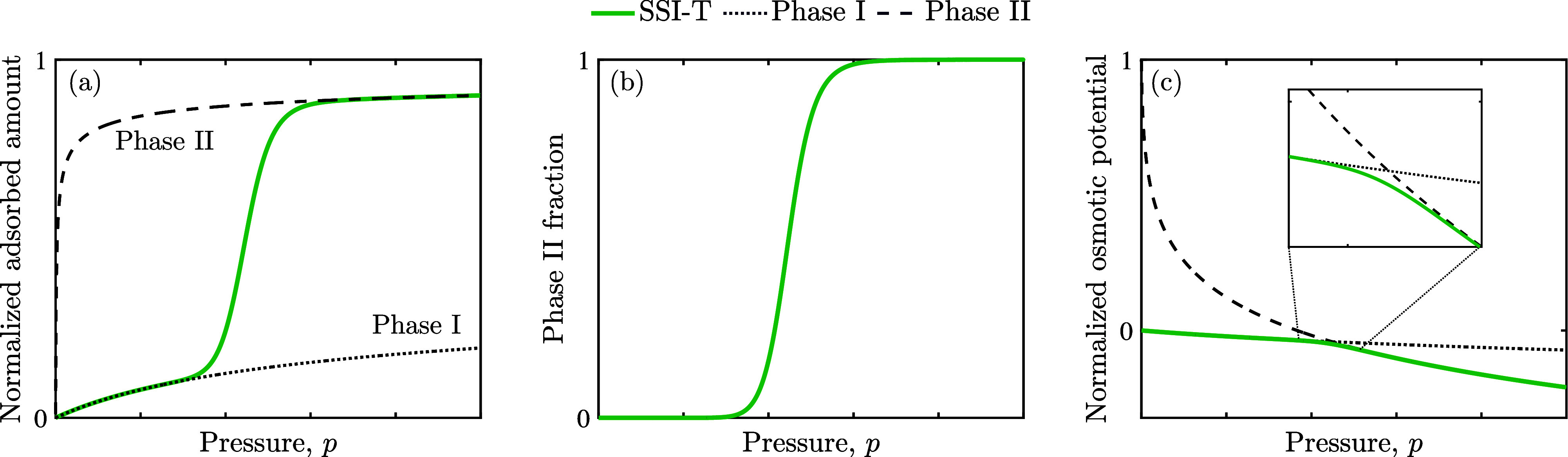
Conceptual illustration of the SSI-T model and its features demonstrated for a framework that exhibits a reversible isotherm and undergoes a single guest-induced transition between a low and high volume phases. (a) Adsorption isotherm for the representative flexible adsorbent (green solid line) and the adsorption isotherms for the two hypothetical phases I and II (dotted and dashed black lines, respectively) as a function of pressure for a single adsorbate. (b) Phase diagram for the same isotherm showing the mole fraction of Phase II present in the adsorbent as a function of pressure. (c) Normalized osmotic potential for the representative flexible adsorbent at the same temperature (green solid line) and the two hypothetical phases I and II (dotted and dashed black lines, respectively) as a function of pressure. The vertical axis is normalized by the free energy difference of the empty host (i.e., *p* → 0) between the two phases. The same profile zoomed around the pressure at which the osmotic potentials of the two phases cross is shown as an inset, highlighting that a smooth and continuous transition is energetically more favorable (a lower free energy path) shown by the green line, compared to an abrupt transition shown by the black lines.

### 2.3 Conditions for Phase Transition: Osmotic Statistical Ensemble

For adsorption-induced transitions, the phase behavior of the host can be described by the osmotic statistical ensemble, eq 1, which relates the free energy of the host framework *F* ^host^(*T*) to the grand potential of the adsorbed phase Ω^ads^ (host–guest interactions) and the guest–guest interactions.^23,36^ The first term in eq 1 describes the grand thermodynamic potential of the adsorbed phase Ω^ads^(*v*_uc_, *p*, *T*), obtained through isothermal integration of the adsorption isotherm, where *N* ^I^ and *N* ^II^ are the number of adsorbed molecules in each phase described by a continuous isotherm and *V*_*m*_(*p*, *T*) is the molar volume of the pure sorbate. For a system undergoing a transition between two rigid phases, *N* ^ads^ is modeled using the SSI (eq 4) as illustrated by the dotted and dashed black lines in Figure 2a. However, note that *v*_uc_ in eq 1 corresponds to the unit cell volume, including the volume of the solid, as opposed to the void volume *v* used in the SSI. The second term in eq 1, *pv*_uc_, describes guest–guest interactions within the cavity and can often be neglected as its magnitude at relevant pressures is negligible compared to the other terms. The third term, *F* ^host^(*T*), describes the contributions to the osmotic potential from the free energy of the host. This contribution is further separated into two parts: (1) the free energy of the empty host (*in vacuo*) *F*_0_^host^(*v*_uc_,*T*) for which the temperature dependence is given by *F*_0_^host^(*v*_uc_,*T*) = *U*_0_^host^-*TS*_0_^host^, where the internal energy and entropic terms are both dependent on *v*_uc_, and (2) the elastic free energy contribution to the osmotic potential Ω^stress^(*v*_uc_, *p*, *T*), arising from volumetric stress exerted on the framework due to adsorption, described by the bulk modulus *k*_*v*_ and volumetric strain ε. This final term can be used to predict hysteresis in materials that undergo “breathing” transitions.^24,37,38^

For a guest-induced transition to occur between two phases, the difference in osmotic potential between the two phases, ΔΩ^os^ = Ω_II_^os^(*p*_tr_,*T*)-Ω_I_^os^(*p*_tr_,*T*), at the transition pressure *p*_tr_ must be zero. Taking phase II as the reference state (*v*_uc_^I^ < *v*_uc_^II^) and assuming the bulk phase is ideal, the osmotic potential difference can be approximated as follows

8 $$\begin{array}{l} \Delta \Omega^{os}\left(v_{uc},p,T\right)=\Delta F_{0}^{host}\left(v_{uc},T\right)+\Delta \Omega^{stress}\left(v_{uc},p,T\right)+p\Delta v_{uc}-[\int_{0}^{p_{tr}}N^{II}\left(v_{uc}^{II},P,T\right)V_{m}\left(P,T\right)dP\\ -\int_{0}^{p_{tr}}N^{I}\left(v_{uc}^{I},P,T\right)V_{m}\left(P,T\right)dP] \end{array}$$

Assuming negligible guest–guest interactions, *pv*_uc_ ≈ 0, and a reversible system (for which adsorption and desorption branches overlap, i.e., Ω^stress^(*v*_uc_, *p*, *T*) = 0), Δ*F*_0_^host^(*v*_uc_,*T*) can be calculated graphically by computing the difference in Ω^ads^(*v*_uc_, *p*, *T*) between the two phases at the transition pressure, as shown graphically by the difference between the black dashed and dotted lines at the limit of *p* → 0 in Figure 2c.^23^

### 2.4 Phase-Coexistence: Approximating the Canonical Partition Function in the Macroscopic Limit

Applying the analysis presented above, we can describe the conditions for phase transition in terms of the number of transitions and the pressure at which they occur as a function of temperature. While this analysis describes a sharp discontinuous transition at the transition pressure, in most real macroscopic systems (*M* → ∞), the cavities can undergo a phase transition more or less independently, resulting in an intermediate pressure range of phase coexistence around the transition pressure, and a continuous observed isotherm. To derive an analytical isotherm for a macroscopic system based on the assumptions of SSI, we first need to determine the canonical partition function for a single cavity $Z^{trans}$ . However, this is not possible using the same approach as for a system of rigid cavities since the cavities are not strictly identical at all conditions, i.e., during a phase coexistence. We resolve this by using an analogous approach to that used by Hiraide et al.^26^ in their development of the structural transition type adsorption (STA) isotherm.

Taking the statistical ensemble for SSI described previously with an adsorbent consisting of *M* cavities at thermal and chemical equilibrium with an infinite reservoir of heat and particles, we distribute the cavities into two subsystems: all *m* cavities in phase II are in one subsystem and the remaining *M* – *m* cavities in phase I are in the other, such that the osmotic free energy of the system is minimized. As such, at any given state, each of these independent subsystems can be treated as a frozen solid that can be described by the partition function given by eq 2 and the corresponding SSI model. For this system, the probability that *m* cavities exist in phase II at a given state is given by the proportionality relation below.

9 $$\rho^{host}\left(m\right)\propto \left(\frac{M}{m}\right)\exp\left[\left(M-m\right)\frac{-F^{I,host}\left(T\right)}{kT}\right]\exp\left[m\frac{-F^{II,host}\left(T\right)}{kT}\right]\propto \left(\frac{M}{m}\right){\left(\exp\left[\frac{-\Delta F^{host}\left(T\right)}{kT}\right]\right)}^{m}$$

Taking the extreme limits on *M*, when M = 1 (i.e., a single cavity consisting of the entire pore volume of the entire adsorbent), the osmotic free energy of the system is minimized at the transition point by undergoing a discontinuous phase transition. At the macroscopic limit, *M* ≫ 1, the osmotic potential of the system is minimized by allowing for both phases to coexist within a range of pressures around the pressure at which the osmotic free energy of an isolated cavity would transition to phase II, as shown in the insets in Figure 2c)

Following this, we can derive the canonical partition function for the macroscopic system $Q^{trans}$ by summing, over all values of *m*, the product of the probability relation (eq 9) with the grand partition function for all the cavities in each bin ( ${\left[Z^{I}\right]}^{M-m}$ and ${\left[Z^{II}\right]}^{m}$ , described by eq 2). Upon rearranging, we obtain an expression for the ensemble average state of a single cavity $Z^{trans}$ .

10 $$Q^{trans}={\left(Z^{trans}\right)}^{M}=\sum_{m=0}^{M}\rho^{host}\left(m\right){\left[Z^{I}\right]}^{M-m}{\left[Z^{II}\right]}^{m}=\sum_{m=0}^{M}\left(\frac{M}{m}\right){\left(\exp\left[\frac{-\Delta F^{host}\left(T\right)}{kT}\right]\right)}^{m}{\left(\sum_{n=0}^{\omega^{I}}Z^{I}\left(n\right)a^{n}\right)}^{M-m}{\left(\sum_{l=0}^{\omega^{II}}Z^{II}\left(l\right)a^{l}\right)}^{m}={\left[\left(\sum_{n=0}^{\omega^{I}}Z^{I}\left(n\right)a^{n}\right)+\exp\left[\frac{-\Delta F^{host}\left(T\right)}{kT}\right]\left(\sum_{l=0}^{\omega^{II}}Z^{II}\left(l\right)a^{l}\right)\right]}^{M}$$

here, the configurational partition functions (*Z* ^I^ or *Z* ^II^) for adsorbed molecules in each cavity are derived in the same way as that for a rigid framework (eq 3). Note that for this derivation, we return to using the void volume of the cavities *v* as a reference state volume for calculating thermodynamic properties, and as such, the free energy difference is calculated for that volume. For comparison with literature sources, these properties can be converted from a volume basis to a mass basis using the same approach given by eq 5.

### 2.5 Simplified Statistical Isotherm for Stimuli-Responsive Materials

Having obtained an analytical approximation for $Z^{trans}$ , we obtain an analytical expression for the adsorbed amount in a stimuli-responsive material given by the SSI-T isotherm ${\left\langle N\right\rangle }_{\theta }^{SSI‐T}$ .

11 $${\left\langle N\right\rangle }_{\theta }^{SSI‐T}\left(p,T\right)=\frac{p}{Z^{trans}}\frac{\partial Z^{trans}}{\partial p}=\left(1-x_{\theta }\right){\left\langle N\right\rangle }^{I}+x_{\theta }{\left\langle N\right\rangle }^{II},,\theta =\left\{ads,des\right\}$$

here, the subscript θ distinguishes between the adsorption and desorption branches, and *x*_θ_ is the fraction of cavities in phase II, which is a continuous function of *p* and *T* and is given by

12 $$x_{\theta }\left(p,T\right)=\frac{\frac{\sum_{l=0}^{\omega^{II}}\frac{{\left(K^{II}\left(T\right)p\right)}^{l}}{l!}\left(\frac{1-lb/v^{II}}{1-b/v^{II}}\right)}{\sum_{n=0}^{\omega^{I}}\frac{{\left(K^{I}\left(T\right)p\right)}^{n}}{n!}\left(\frac{1-nb/v^{I}}{1-b/v^{I}}\right)}\exp\left[\frac{-\Delta F_{\theta }^{host}\left(T\right)}{kT}\right]}{1+\frac{\sum_{l=0}^{\omega^{II}}\frac{{\left(K^{II}\left(T\right)p\right)}^{l}}{l!}\left(\frac{1-lb/v^{II}}{1-b/v^{II}}\right)}{\sum_{n=0}^{\omega^{I}}\frac{{\left(K^{I}\left(T\right)p\right)}^{n}}{n!}\left(\frac{1-nb/v^{I}}{1-b/v^{I}}\right)}\exp\left[\frac{-\Delta F_{\theta }^{host}\left(T\right)}{kT}\right]}=\frac{\frac{Z^{II}}{Z^{I}}\exp\left[\frac{-\Delta F_{\theta }^{host}\left(T\right)}{kT}\right]}{1+\frac{Z^{II}}{Z^{I}}\exp\left[\frac{-\Delta F_{\theta }^{host}\left(T\right)}{kT}\right]}$$

where, the terms ${\left\langle N\right\rangle }^{I}$ and ${\left\langle N\right\rangle }^{II}$ in eq 11 describe the hypothetical isotherms for the two rigid phases described by the original SSI (eq 4), shown as dotted and dashed lines in Figure 2a for the two phases. The derivation of eq 11 is given in the Supporting Information.

At *p* and *T*, these two isotherms are weighted according to the probability of their occurrence by the expected fraction of cavities in phase II, *x*_θ_ (Figure 2b), which are equivalent for adsorption and desorption branches for a reversible isotherm, i.e., ΔΩ^stress^ = 0 (green solid lines Figure 2). In this formulation, the expression for the expected value for the phase fraction is a function of the canonical partition functions for the two hypothetical phases at the given state ( $Z^{I}$ and $Z^{II}$ ), and so is influenced by the isotherms for each phase in addition to the free energy barrier Δ*F*_θ_^host^(*T*). The temperature dependence of *x*_θ_ can be used to generate a continuous phase diagram for each host–guest system, and this is illustrated in Figure 3 for the hypothetical system illustrated in Figure 2.

**Figure 3:**
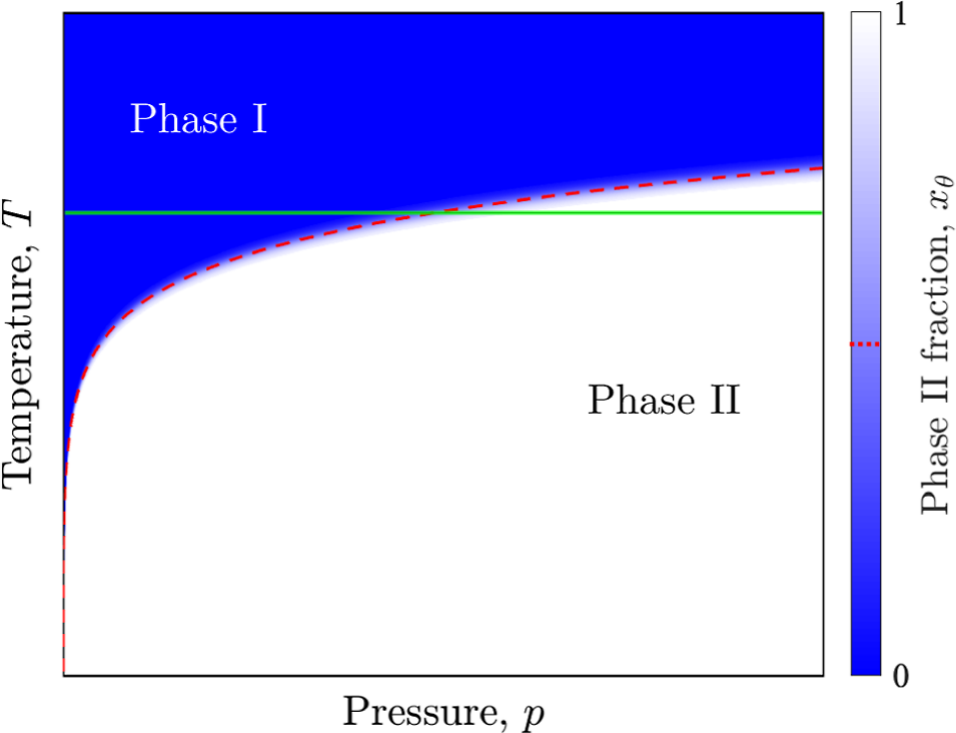
Phase diagram for the adsorption of a single adsorbate in the representative flexible adsorbent system illustrated in Figure 2 for the temperature given by the green horizontal line, showing the molar fraction of phase II as a function of pressure and temperature. The phase fraction is represented by a continuous gradient from blue to white and is obtained by the solution of eq 12.

### 2.6 History-Dependence and Adsorption Hysteresis: Approximating the Osmotic Contribution from Guest-Induced Stress

For systems that exhibit hysteresis or multiple transitions at constant temperature (e.g., breathing transitions in MIL-53­(Al)^39,40^ )—i.e., ΔΩ^stress^ ≠ 0—we model the elastic free energy contribution to the osmotic potential difference as a constant energy barrier ΔΩ^stress^, as proposed by Triguero et al.,^41^ which is treated as an adjustable parameter that can be estimated by fitting the model to adsorption and desorption isotherms simultaneously. Using the same basis as above and taking phase II as the reference state, transitions from phase I to II result in this parameter being positive (as is the volumetric strain during expansion), requiring a greater barrier to be overcome, and negative with equal magnitude for a reverse transition from phase II to I. Equation 11 describes the major branches of either the adsorption ${\left\langle N\right\rangle }_{ads}^{SSI‐T}$ or desorption isotherm ${\left\langle N\right\rangle }_{des}^{SSI‐T}$ as shown by red and blue solid lines in Figure 4, respectively, now assuming a nonzero contribution to the osmotic free energy from adsorption induced stress.

**Figure 4:**
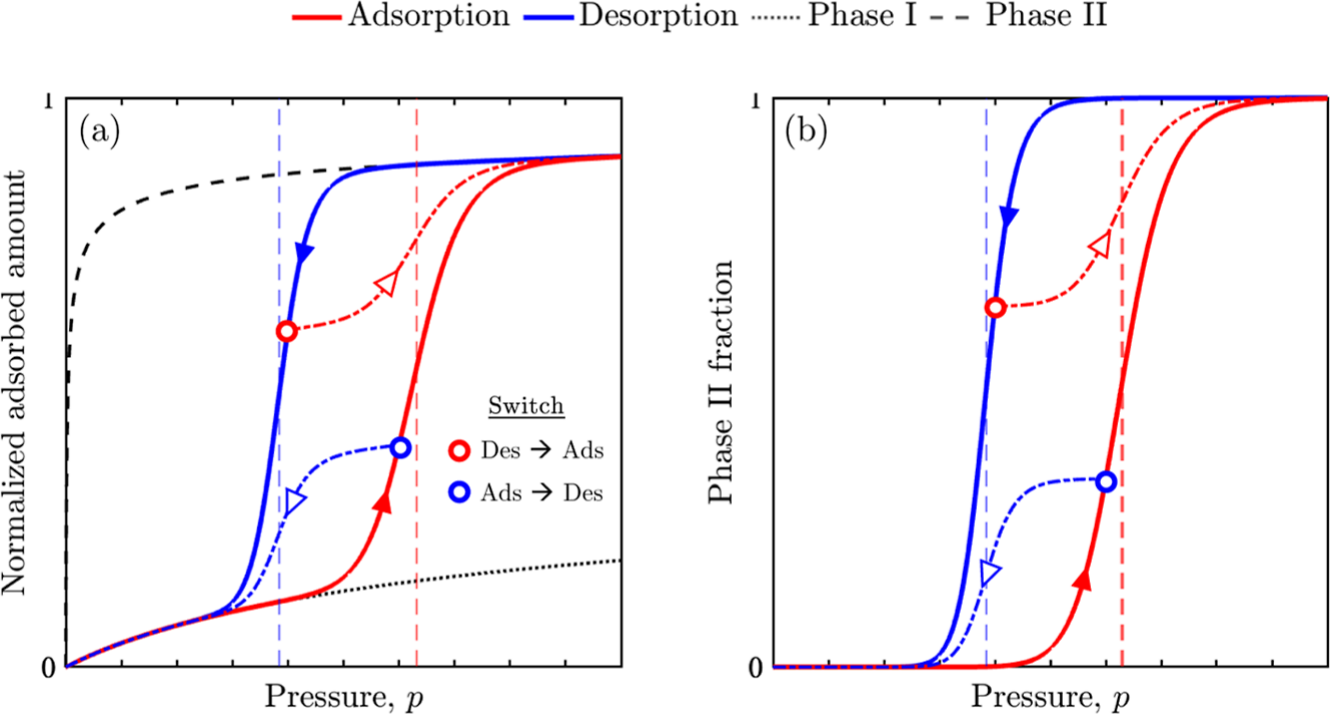
Conceptual illustration of the SSI-T model and its features demonstrated for a framework that exhibits hysteresis and undergoes a single guest-induced transition between a low and high volume phase. (a) Adsorption (red) and desorption (blue) isotherms for the representative flexible adsorbent and the adsorption isotherms for the two hypothetical phases I and II (dotted and dashed black lines, respectively) as a function of pressure for a single adsorbate. The solid lines correspond to the major hysteresis loop given by eq 11, and the dotted–dashed lines correspond to minor hysteresis curves showing complete reversal from a state of partial transition on the major loop to the opposite branch on the major loop given by eqs S20 and S21. (b) Phase diagram for the same isotherms showing the mole fraction of Phase II present in the adsorbent as a function of pressure for the major and minor loops.

The description of the model presented thus far describes the major hysteresis loops; however, the model is discontinuous if the *direction* of transition is switched at conditions where the system is partially transitioned. To eliminate this issue, we propose a scaling method (similar to those used to describe magnetic hysteresis^42^) to predict the history-dependence of phase-behavior, i.e., to compute the correct and continuous equilibrium path within the hysteresis loop without any additional assumptions. This is shown in Figure 4 as dotted–dashed lines and described in detail in the Supporting Information.

### 2.7 Choice of Cavity Volume and Cell Interactions: Effective Domain Size

A key point to note is that the assumption of noninteracting cavities used in arriving at this model formulation is not valid for many adsorbents when dealing with phase transitions. In most of these systems, neighboring cavities do interact in different manners simply due to the connected nature of unit cells within a *crystallite*, and a structural transition in one will affect the free energy of the neighbors. Examples of such interacting systems include MOFs that share rotating ligands,^43,44^ and layered frameworks that undergo shear-based transitions.^41^ Developing an explicit model that can describe guest-induced phase transitions in a system consisting of interacting unit cells is not possible as long as the interactions between cavities need to be modeled mechanistically for different types of transitions,^45^ generally requiring numerical or implicit solution of the model equations.^46^

Instead of defining each subsystem as a unit cell, we therefore use the concept of an *effective domain*, which, from a *macroscopic perspective*, can be defined as the smallest unit of the adsorbent that can independently undergo a phase transition. Mathematically, this is defined as some multiple (η) of unit cells for which the accessible (or void) volume for adsorption in each phase is given by *v* ^I^ = η*v*_uc_^I^ and *v* ^II^ = η*v*_uc_^II^. The void volume of a unit cell can be obtained directly from crystallographic pore information. When crystallographic information is unavailable, *v*_uc_^II^ can be calculated by taking phase II as the reference state, by using the micropore volume *v*_mic_^II^ and the molar mass of a unit cell *M*_uc_ as follows.

13 $$v_{uc}^{II}=\frac{v_{mic}^{II}M_{uc}}{N_{A}}$$

This results in a model formulation where the domain size η is an adjustable parameter that can be fitted to match experimentally observed transition behavior. Additionally, this assumption generalizes the model to any system that undergoes guest-induced transitions regardless of the mechanism involved or the geometry of the framework. For instance, whether the effective domain is a single crystal, a layer of unit cells within a crystal, or a single unit cell, the phase transition behavior is determined by state variables and thermodynamic properties defined per mole of adsorbent.

### 2.8 Predicting Mixture Adsorption in Flexible Adsorbents

The extension of the SSI-T isotherm to mixtures follows directly from that for the frozen solid model (eq 7), and the above derivation of the SSI-T isotherm for a single adsorbate. The equation for the adsorbed amount of component α in the presence of a second adsorbate β at equilibrium is thus given by

14 $${\left\langle N\right\rangle }_{\alpha \beta }^{SSI‐T}=\left(1-x_{\alpha \beta }\right){\left\langle N\right\rangle }_{\alpha }^{I}+x_{\alpha \beta }{\left\langle N\right\rangle }_{\alpha }^{II}$$

where ${\left\langle N\right\rangle }_{\alpha }^{I}$ and ${\left\langle N\right\rangle }_{\alpha }^{II}$ are the hypothetical competitive SSI isotherms for component α in each phase. As in eq 11, these two isotherms are weighted by the expected phase fraction *x*_αβ_, which for a binary mixture is given by

15 $$x_{\alpha \beta }=\frac{\frac{Z_{\alpha \beta }^{II}}{Z_{\alpha \beta }^{I}}\exp\left[\frac{-\Delta F_{\theta }^{host}\left(T\right)}{kT}\right]}{1+\frac{Z_{\alpha \beta }^{II}}{Z_{\alpha \beta }^{I}}\exp\left[\frac{-\Delta F_{\theta }^{host}\left(T\right)}{kT}\right]}$$

where the ratio of canonical partition functions in the expression for *x*_αβ_ uses the definition in eq 6 for the competitive SSI model.

### 2.9 Parameter Estimation

The SSI-T isotherm for a single adsorbing gas is described by ten parameters for a system that exhibits hysteresis and nine for one that does not. These are the void volumes of the effective domain in the two phases *v* ^I^ and *v* ^II^, free-energy difference between the two empty hosts given by Δ*U*_0_^host^ and Δ*S*_0_^host^ (and ΔΩ^stress^ if the isotherms are hysteretic or contain multiple transitions), and five parameters to describe the SSI isotherms of the hypothetical rigid structures of the two phases. Here, we can eliminate one parameter by assuming the effective molecular volume *b* remains unchanged between transitions.

In our previous works, we presented a methodology for parametrizing experimental equilibrium data using various models, including the SSI model.^34,47–49^ Here, we extend this methodology to account for the relative complexity of the SSI-T model, thereby accurately predicting sorbate-independent parameters and ensuring the best fit to the available data. Additionally, the model describes absolute adsorption; hence, any experimental isotherms are converted from excess to absolute by assuming a constant density of the adsorbed phase (given by the sorbate’s liquid density at the triple point) before any parameter estimation. Following this, the parameter estimation to parametrize the adsorption and desorption equilibria in a single host for multiple sorbates is carried out in the following 3 steps:

1. First, the available data (measured at at least 2 temperatures to determine temperature-dependent parameters) is split into the two phases based on approximate threshold values of adsorbed amount, and neglecting any data points within an apparent coexistence region. The two sets of data are then used to independently parametrize the hypothetical isotherms for each phase using the SSI model to determine $K_{0}^{I}$ , $K_{0}^{II}$ , Δ*u*_ads_^I^ and Δ*u*_ads_^II^ to describe the Henry’s law constant (host–guest interactions), the effective molecular volume of the sorbate *b*, and the ratio of domain volumes between the two phases *v* ^I^/*v* ^II^. In this step of the parameter estimation, the cavity volume of phase II (*v* ^II^) is assigned an arbitrary nominal value of 2000 Å^3^/cavity as the scaled isotherms for each phase are mostly insensitive to the magnitude of the void volume as shown in the black lines in Figure 12, and the true volume is estimated in the next step.
2. In the second step these six parameters are fixed, and all of the available data points for the sorbate including those points in the coexistence regions and desorption isotherms (if hysteresis is observed) are used to estimate the remaining structural parameters Δ*U*_0_^host^, Δ*S*_0_^host^, ΔΩ^stress^, and the true void volume of the effective domain *v* ^II^.
3. The five structural parameters (the four obtained in step 2, along with *v* ^I^/*v* ^II^ from step 1) are sorbate-independent for a given material, and therefore are subsequently fixed to estimate the five remaining sorbate-dependent parameters for each additional sorbate.

The estimation of the parameters in steps 1 and 3 is carried out using a maximum likelihood estimator (MLE) approach.^47,48^ The procedure involves minimizing the objective function for the MLE *J*(**θ**) that is given as follows

16 $$J\left(\theta \right)=\frac{N_{t}}{2}\ln\left(\sum_{j=1}^{N_{t}}{\left({\left\langle N\right\rangle }^{\exp}-{\left\langle N\right\rangle }^{model}\left(\theta \right)\right)}^{2}\right)$$

where *N*_t_ is the total number of data points for a pure component at all temperatures, ${\left\langle N\right\rangle }^{\exp}$ is the vector containing the absolute adsorbed amount data in units of molec. cavity^–1^, and ${\left\langle N\right\rangle }^{model}\left(\theta \right)$ is the corresponding calculated value of the absolute amount adsorbed at the same *p* and *T* for a set of isotherm parameters given by the vector **θ** using either the SSI or the SSI-T for step 1 and 3, respectively. The objective function is minimized using the built-in MATLAB function globalsearch MATLAB R2023a (The Mathworks Inc., United States) with fixed upper and lower bounds set for each of the parameters, which are summarized in the Supporting Information. The procedure for estimating the SSI-T parameters starting from experimental unary excess isotherm data is summarized in Figure 5.

**Figure 5:**
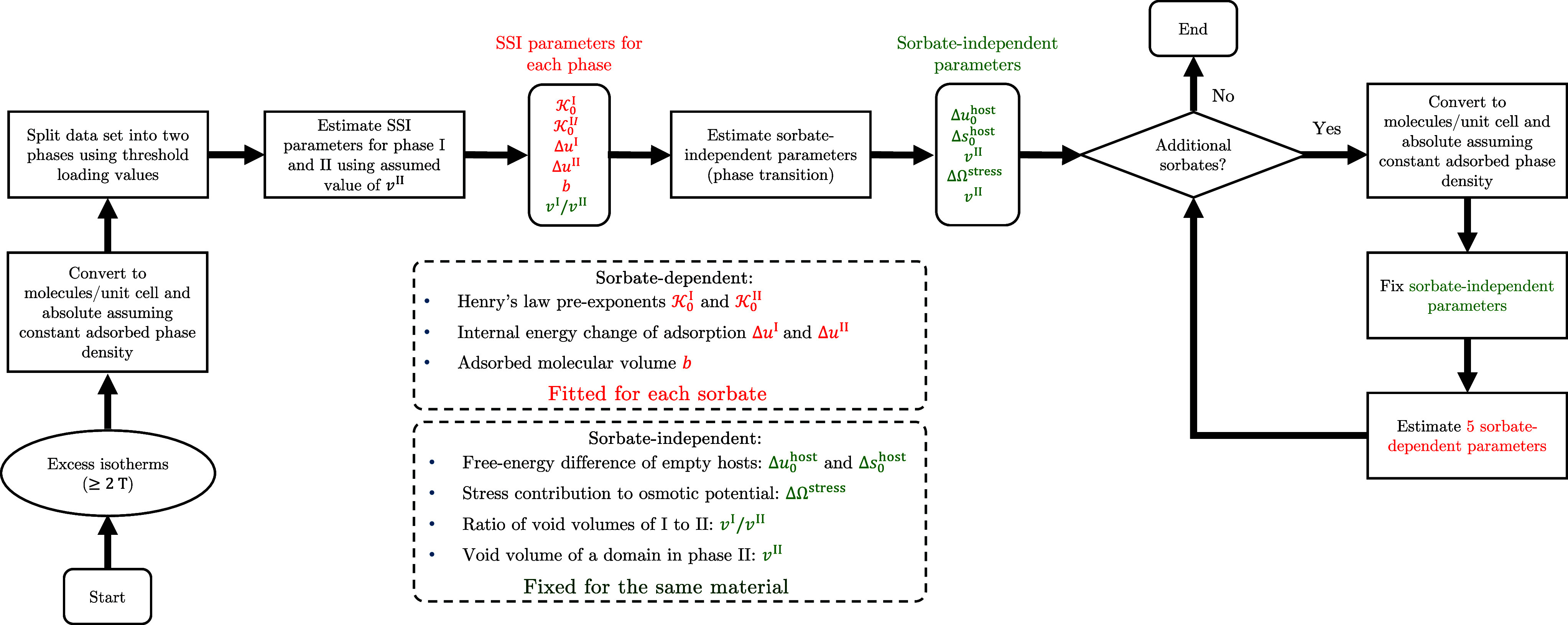
Schematic workflow of the procedure used to estimate the model parameters for the SSI-T model for a given experimental data set. The parameters denoted in red are sorbate-dependent parameters and need to be estimated for every species, and those in green are sorbate-independent, which are fixed across species for a given material.

## 3 Results and Discussion

In this section, we demonstrate the validity of the SSI-T model for describing adsorption equilibria in flexible materials by applying it to unary and binary isotherm data from experimental literature across a range of systems. Following this, we also compare the SSI-T model with a previously reported isotherm for flexible adsorbents from the literature. We then discuss the key features of the proposed model, namely the concept of an effective domain that directly relates to unit cell properties, followed by a discussion on the state-dependence of the isosteric enthalpy of adsorption in flexible adsorbents and its potential implications to adsorption-based applications.

### 3.1 Unary Equilibrium Parameterization

In this section, we apply the SSI-T model to two data sets containing equilibrium isotherms at a broad range of pressure, temperature, and adsorbates—ZIF-7 ^28^ and MIL-53­(Al)^40,50^ —to validate the key assumptions used in the development of the model. We consider the agreement between model predictions and experimental data, with a focus on the inherent temperature dependence of the model, fixed sorbate-independent parameters, and the relationships between estimated parameters and physical properties of the adsorbents.

#### 3.1.1 Gate-Opening Transitions in ZIF-7

To validate the methodology, the SSI-T model was fitted to experimental adsorption and desorption isotherm data for CO_2_, C_2_H_6_, N_2_, CH_4_, and Ar in ZIF-7 reported by Yang et al.^28^ The fitted model predictions, alongside the experimental data, are shown in Figure 6, and the corresponding model parameters are given in Table 1.

**Figure 6:**
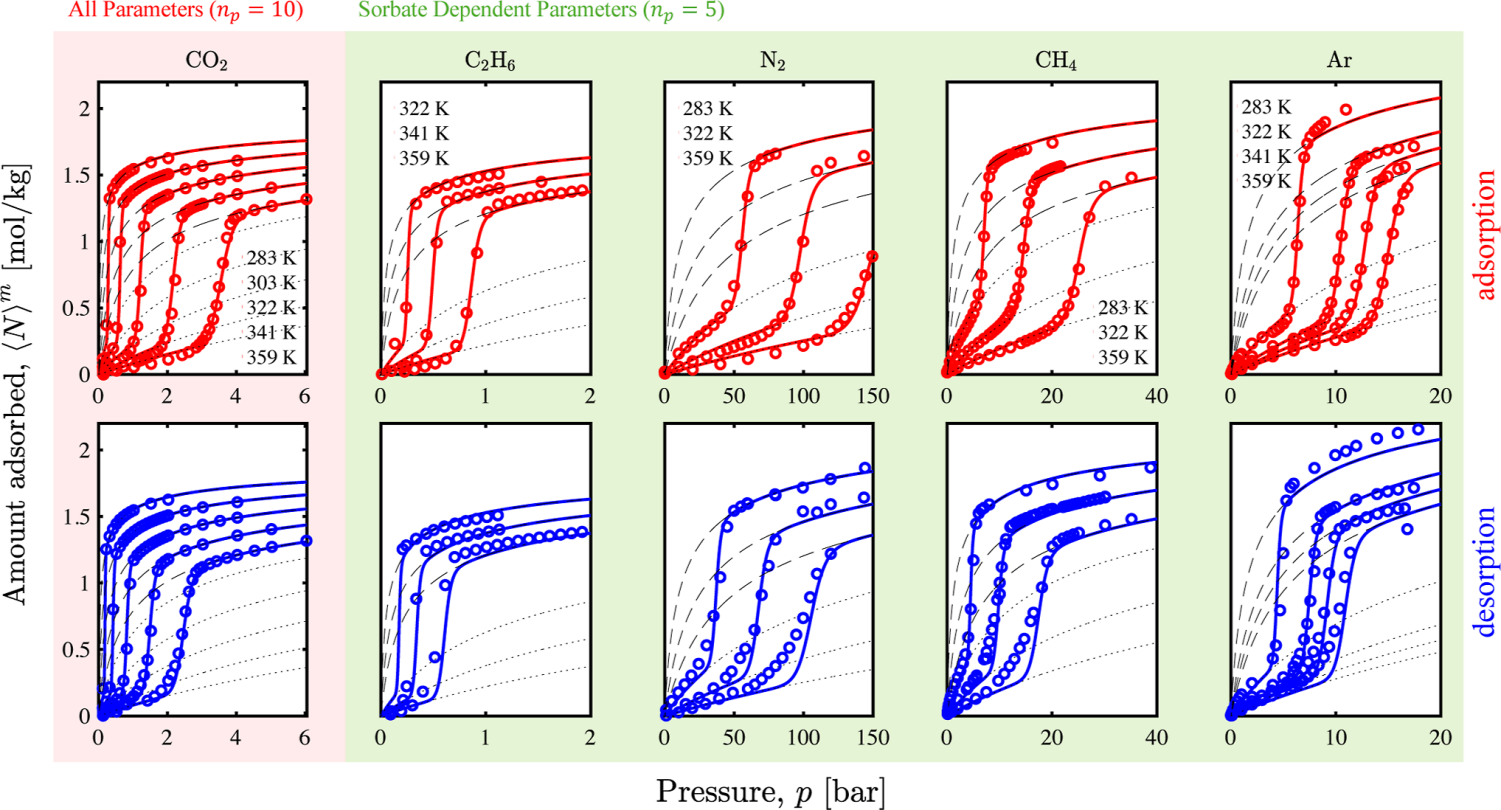
Absolute adsorption (top row, red) and desorption (bottom row, blue) isotherms of CO_2_, C_2_H_6_, N_2_, CH_4_, and Ar on ZIF-7 as a function of pressure at various temperatures (labeled in the insets). The empty symbols correspond to equilibrium data reported by Yang et al.,^28^ and the solid lines represent the SSI-T model parametrization given by eq 11 and the parameters in Table 1. The dotted and dashed black lines correspond to the hypothetical isotherms for phases I and II, respectively. The equilibrium data for CO_2_ were first used to fully parametrize the SSI-T model to obtain the corresponding sorbate-dependent parameters for CO_2_ and sorbate-independent parameters for ZIF-7. The sorbate-independent parameters were then fixed in estimating the sorbate-dependent isotherm parameters for the remaining sorbates.

**Table 1:** SSI-T Model Parameters Estimated to Describe the Adsorption and Desorption Equilibria of CO_2_, C_2_H_6_, N_2_, CH_4_, and Ar on ZIF-7^a^ ^,^ ^b^ ^51^

ZIF-7					
**Parameter**	**CO**_**2**_	**C**_**2**_ **H**_**6**_	**N**_**2**_	**CH**_**4**_	**Ar**
	**Phase I (Closed)**				
$K_{0}^{I}$ [molec. cavity^–1^ bar^–1^]	9.16 × 10^–5^	8.42 × 10^–6^	1.77 × 10^–4^	4.66 × 10^–4^	7.60 × 10^–3^
-Δ*u*_ads_^I^[kJ mol^–1^]	29.70	40.21	17.73	20.78	13.63
*b* ^I^ [Å^3^/molec.]	70.0	65.4	52.2	55.6	45.0
	**Phase II (Open)**				
$K_{0}^{II}$ [molec. cavity^–1^ bar^–1^]	4.84 × 10^–4^	3.51 × 10^–4^	2.53 × 10^–3^	4.94 × 10^–3^	5.30 × 10^–2^
-Δ*u*_ads_^II^[kJ mol^–1^]	34.19	39.19	17.70	21.35	15.17
*b* ^II^ [Å^3^/molec.]	*b* ^I^				
	**Structural parameters**				
*v* ^I^ [Å^3^/cavity]	3271				
*v* ^II^ [Å^3^/cavity]	*v* ^I^				
Δ*U*_0_^host^[kJ mol^–1^]	143.71				
Δ*S*_0_^host^[J mol^–1^ K^–1^]	31.26				
ΔΩ^stress^ [kJ mol^–1^]	10.98				
effective domain size [-]	2 unit cells of void volume 1675 Å^3^ ^51^				

a Structural parameters Δ*U*_0_^host^, Δ*S*_0_^host^, and ΔΩ^stress^ are reported per mole of cavities.

b The effective domain size for the ZIF-7 data reported by Yang et al.^28^ was estimated to be approximately equal to 2 unit cells of void volume 1675 Å^3^.

The model reproduces transition pressures (and steepness), hysteresis width, and phase fractions across temperatures for all 5 sorbates. Here, steps 1 and 2 of the parameter estimation outlined in Section 3 were carried out for the data on CO_2_, yielding five sorbate-dependent parameters for CO_2_ and the five sorbate-independent structural parameters for ZIF-7. Step 3 was repeated for each additional sorbate to estimate the sorbate-dependent parameters. The resulting fits to the experimental data for the remaining 4 sorbates demonstrate the universality of structural (sorbate-independent) parameters, which is a feature that is not present in previously reported isotherm models such as the LJMY model^28^ and the STA model.^26^ The accurate prediction of the hysteresis in the desorption data also validates the assumption of using a constant value for the contribution to the osmotic potential difference from adsorption-induced stress ΔΩ^stress^.

The estimated parameters can be used to elucidate some insights into the mechanism of adsorption and phase transition in each of the two phases and the nature of the phase transition. First, we note that the parameter estimation results in equal values for the void volume of the effective domain for the two phases, i.e., *v* ^I^ = *v* ^II^, which is expected for gate-opening transitions,^52^ which is understood to be the mechanism of transition for ZIF-7. Additionally, the effective domain on average consists of 2 unit cells, suggesting significant interactions between adjacent unit cells of the ZIF-7 samples (particle size of 70 μm) used in these measurements. As a result of these interactions, we see a relatively sharp transition from the closed to the open phase. From the perspective of material formulation, it has also been shown that the sharpness of this transition and the transition conditions in ZIF-7 are influenced by the size and shape of the particles. This is apparent when we compare the data in Figure 6 to CO_2_ isotherms on different ZIF-7 samples of smaller particle sizes reported by Cai et al.,^53^ which show a much more gradual transition. This suggests that the concept of effective domain size may be linked to physical properties of the crystals, such as their shape and size. From the sorbate-dependent parameters, we see that the estimations of internal energy change due to adsorption Δ*u*_ads_ for the two phases are approximately equal for all sorbates aside from CO_2_. Given that the effective molecular volume *b* is conserved between phases, this indicates that the difference in adsorbed amount between the two phases for these sorbates is a consequence of a change in the pre-exponential factors $K_{0}$ , which reflects a change in the entropic barriers to adsorption upon a gating transition.^54^ Accordingly, we observe at least an order of magnitude increase in $K_{0}$ from the closed to open phases, and for CO_2_ we also see an increase in the magnitude of −Δ*u*_ads_ of about 4.5 kJ mol^–1^.

#### 3.1.2 Breathing Transitions in MIL-53­(Al)

Figure 7 shows the experimental unary adsorption and desorption isotherms for CO_2_, CH_4_, and Xe in MIL-53­(Al) from Boutin et al.^40,50^ and the corresponding model fits. Here, the sorbate-independent structural parameters were obtained by fitting the model to the three CO_2_ isotherms that show the two transitions, measured at 254, 273, and 298 K. The estimated model parameters for the adsorption of CO_2_, CH_4_, and Xe in MIL-53­(Al) are given in Table 2. Overall, the SSI-T model describes the equilibrium data accurately, particularly for the isotherms used to parametrize the data (shown with green temperature labels in Figure 7). The hypothetical isotherms for each of the phases (black dashed and dotted lines) consistently fit the experimental data, and the phase transition behavior is replicated with respect to the transition pressures and the extent of hysteresis. The phase transition behavior is represented on a continuous phase diagram to the right of the isotherms in Figure 7 for all 3 sorbates, with the experimentally determined phase boundaries shown in green.^40,50^ In MIL-53­(Al), the framework is stable in its large pore (LP) phase *in vacuo* and can undergo transitions to narrow-pore (NP) with low adsorbed amounts and back to LP with more adsorption.^55^ These successive transitions are caused when threshold stresses on the framework are overcome in addition to the free energy barrier, which additionally leads to hysteresis in each of the transitions. Previous Langmuir parametrizations of the data shown in Figure 7 indicate that the ratio of maximum adsorption capacity between the NP and LP phases can vary between adsorbates, which, for a fixed framework, suggests a change in the effective volume of adsorbed molecules (molecular configuration) upon breathing transitions.^40,50^ To account for these additional complexities, we made the following adjustments to the model parametrization and solution procedure. First, the constraint on the effective molecular volume *b* between the two phases was relaxed, such that adsorbed molecules can exhibit different effective volumes in each phase, to allow for different adsorbates to be modeled accurately using the same structural parameters. This supposed difference in confinement effects of the adsorbate molecules in the two phases has been directly observed in flexible adsorbents, for instance, in the case of Xenon adsorption in ZIF-4.^56^ Additionally, multiple successive transitions imply a change in the direction of transition along the pressure axis (i.e., LP to NP to LP), and therefore, require a change in the sign of the contribution of adsorption-induced stress to the osmotic free energy ΔΩ^stress^. For such a system, a correct representation of the adsorption isotherms is achieved by using the same methodology used to model partial reversal curves in the methods section (and in the Supporting Information) to model the change in the sign of ΔΩ^stress^ between successive transitions.

**Figure 7:**
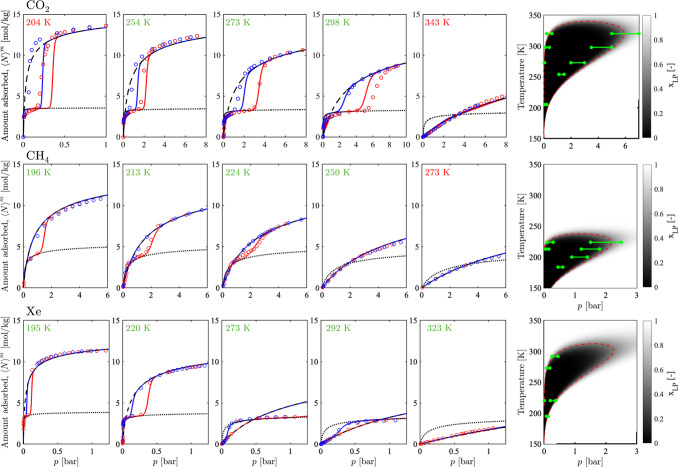
Absolute adsorption (red) and desorption (blue) isotherms of CO_2_, CH_4_, and Xe on MIL-53­(Al) as a function of pressure at various temperatures (labeled in the insets) along with the phase diagrams for each sorbate as a function of pressure and temperature (right column). The empty symbols correspond to equilibrium data reported by Boutin et al.^40,50^ and the solid lines represent the SSI-T model parametrization given by eq 11 and the parameters in Table 2. The dotted and dashed black lines correspond to the hypothetical isotherms for phases I and II, respectively. The equilibrium data for CO_2_ at 254, 273, and 298 K were first used to fully parametrize the SSI-T model to obtain the corresponding sorbate-dependent parameters for CO_2_ and the sorbate-independent parameters for MIL-53­(Al). The sorbate-independent parameters were then fixed in estimating the sorbate-dependent isotherm parameters for the remaining sorbates. The isotherms with the temperature labeled in green were used in the parameter estimation, and those labeled in red are predicted using the SSI-T model parametrization. The horizontal green lines on the phase diagrams show the experimentally observed coexistence region.

**Table 2:** SSI-T Model Parameters Estimated to Describe the Adsorption and Desorption Equilibria of CO_2_, CH_4_, and Xe on MIL-53­(Al)^a^ ^,^ ^b^ ^57^

MIL-53(Al)			
**Parameter**	**CO**_**2**_	**CH**_**4**_	**Xe**
	**Phase I (Narrow Pore)**		
$K_{0}^{I}$ [molec. cavity^–1^ bar^–1^]	1.48 × 10^–4^	1.26 × 10^–2^	4.26 × 10^–2^
-Δ*u*_ads_^I^[kJ mol^–1^]	38.91	15.85	21.61
*b* ^I^ [Å3/molec.]	73.2	42.9	63.8
	**Phase II (Large Pore)**		
$K_{0}^{II}$ [molec. cavity^–1^ bar^–1^]	5.86 × 10^–4^	4.35 × 10^–3^	2.49 × 10^–3^
-Δ*u*_ads_^II^[kJ mol^–1^]	25.67	16.03	22.09
*b* ^II^ [Å^3^/molec.]	52.5	52.0	62.7
	**Structural parameters**		
*v* ^I^ [Å^3^/cavity]	1222.9		
*v* ^II^ [Å^3^/cavity]	4200		
Δ*U*_0_^host^[kJ mol^–1^]	108.40		
Δ*S*_0_^host^[J mol^–1^ K^–1^]	570.84		
ΔΩ^stress^ [kJ mol^–1^]	12.35		
effective domain size [-]	5.5 unit cells of LP void volume 768 Å^3^ ^57^		

a Structural parameters Δ*U*_0_^host^, Δ*S*_0_^host^, and ΔΩ^stress^ are reported per mole of cavities.

b The effective domain size for the MIL-53­(Al) data reported by was estimated to be approximately equal to 5.5 unit cells of LP void volume 768 Å^3^.

From the parameters listed in Table 2, we first highlight the trends in the Henry’s law constant K(T) for each phase, which describe the host–guest interactions. We first note that for all 3 sorbates, K(T) is generally higher for the NP phase compared to the LP phase, resulting in the crossing of the hypothetical isotherms for the two phases at a finite pressure, as seen in Figure 7. Looking more closely into $K_{0}$ and −Δ*u*_ads_ which describe the temperature dependence of K(T) , we note that the magnitude of Δ*u*_ads_ is similar between the two phases for CH_4_ and Xe, which is consistent with the findings for ZIF-7, while the magnitude of this parameter for CO_2_ is greater by approximately 13 kJ mol^–1^ in the NP phase compared to the LP phase. Correspondingly, we see a decrease of an order of magnitude in $K_{0}$ from LP to NP for CH_4_ and Xe, and a slight increase for CO_2_. From the structural parameters, we highlight a 71% reduction in cavity volume from LP to NP phases, which is smaller than the 79% reduction obtained through reported by Titov et al.^57^ Additionally, we note an effective domain size of 5.5 unit cells, which for a breathing adsorbent indicates a cooperative transition of unit cells as described by Triguero et al.,^41^ who showed that breathing transitions in MIL-53­(Al) occur through adsorption-induced shear in two-dimensional layers of unit cells. Translating the cavity void volume in the LP phase to 5.5 unit cells results in a total volume (void and framework) of 7720 Å^3^/cavity which is significantly smaller than a typical MIL-53­(Al) crystal, which would typically be in the order of a few micrometers,^58^ further supporting the coexistence of the two phases within a single crystal.

### 3.2 Binary Equilibrium Predictions

To assess the predictive capabilities of the SSI-T model, we used unary parametrizations of multiple contrasting systems, C_2_H_6_/C_3_H_8_ adsorption in COMOC-2,^59^ CO_2_/H_2_O adsorption in CALF-20 (CAU-10H, and Al-Fumarate in Supporting Information),^60–62^ and CO_2_/CH_4_ adsorption in MIL-53­(Al),^63^ to predict the competitive adsorption equilibria and compare these predictions with existing binary equilibrium data.

#### 3.2.1 Binary Adsorption of Ethane and Propane in COMOC-2

COMOC-2 is a vanadium (V^4+^) containing flexible metal–organic framework which undergoes breathing transition upon adsorption, similar to MIL-53­(Al). This material, however, exhibits the narrow pore phase *in vacuo* and has been shown to exhibit high selectivity in light hydrocarbon systems. Figure 8a shows experimental unary adsorption and desorption isotherms for C_2_H_6_ and C_3_H_8_ at 281, 293, and 303 K, as reported by Couck et al.,^59^ and the corresponding fits obtained using the SSI-T model. The parametrization of the model consists of 5 sorbate-dependent parameters for each gas, and 5 sorbate-independent parameters common to both species, as given in Table 3. From the same study, the binary adsorption isotherms measured at 303 K are shown in Figure 8b, again alongside the predicted binary adsorption isotherms computed using the SSI-T model. The experimental isotherms were provided as total mass of adsorbed species per unit mass of adsorbent and are reported at four equilibrium bulk compositions, namely 20, 35, 50, and 75% by weight.

**Figure 8:**
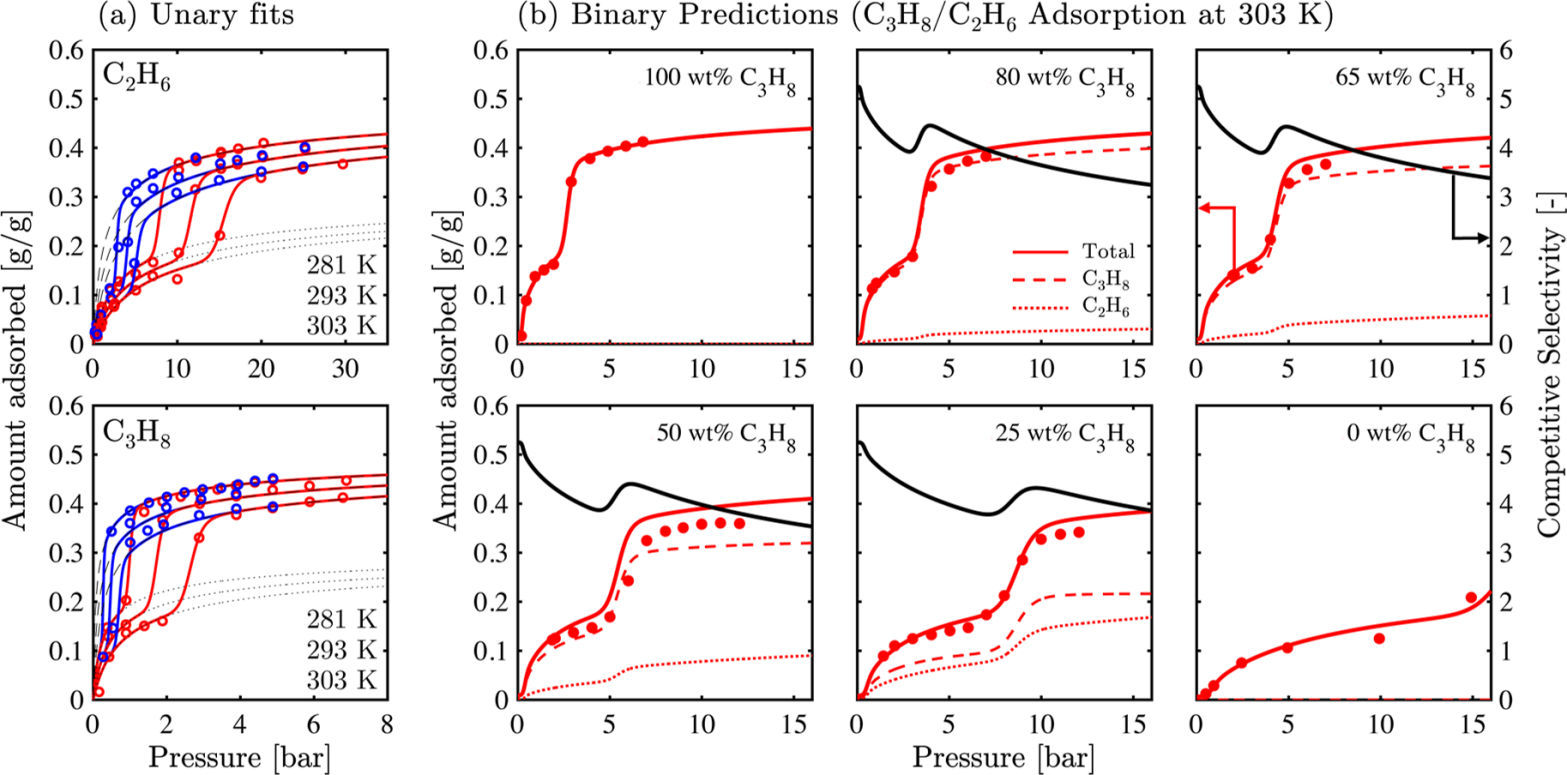
(a) Absolute unary adsorption (red) and desorption (blue) isotherms of C_2_H_6_, and C_3_H_8_ on COMOC-2 as a function of pressure at various temperatures (labeled in the insets). The empty symbols correspond to equilibrium data reported by Couck et al.^59^ and the solid lines represent the SSI-T model parametrization given by eq 11 and the parameters in Table 3. The equilibrium data for C_2_H_6_ were first used to fully parametrize the SSI-T model to obtain the corresponding sorbate-dependent parameters for C_2_H_6_ and the sorbate-independent parameters for COMOC-2. (b) Binary adsorption equilibrium for different mixtures of C_2_H_6_, and C_3_H_8_ in COMOC-2. The vertical axis on the left represents absolute adsorbed amounts, with the filled symbols corresponding to total adsorption equilibrium data reported by Couck et al.^59^ and the solid, dashed, and dotted lines corresponding to SSI-T model predictions computed using eq 14 for total, C_3_H_8_, and C_2_H_6_ respectively. The second vertical axis on the right corresponds to the competitive selectivity of C_3_H_8_ over C_2_H_6_ as a function of pressure for each mixture.

**Table 3:** SSI-T Model Parameters Estimated to Describe the Adsorption and Desorption Equilibria of C_2_H_6_, and C_3_H_8_ on COMOC-2

COMOC-2		
**Parameter**	**C**_**2**_ **H**_**6**_	**C**_**3**_ **H**_**8**_
	**Phase I (Narrow Pore)**	
$K_{0}^{I}$ [molec. cavity^–1^ bar^–1^]	4.70 × 10^–4^	1.22 × 10^–5^
-Δ*u*_ads_^I^[kJ mol^–1^]	24.04	37.42
*b* ^I^ [Å^3^/molec.]	48.2	73.1
	**Phase II (Large Pore)**	
$K_{0}^{II}$ [molec. cavity^–1^ bar^–1^]	9.07 × 10^–5^	2.05 × 10^–6^
-Δ*u*_ads_^II^[kJ mol^–1^]	31.05	45.84
*b* ^II^ [Å^3^/molec.]	*b* ^I^	
	**Structural parameters**	
*v* ^I^ [Å^3^/cavity]	2743	
*v* ^II^ [Å^3^/cavity]	4287	
Δ*U*_0_^host^[kJ mol^–1^]	369.1	
Δ*S*_0_^host^[J mol^–1^ K^–1^]	847.7	
ΔΩ^stress^ [kJ mol^–1^]	27.98	

The parametrization of the SSI-T model with the unary data for the two sorbates yields an accurate description of the experimental data for both adsorption and desorption. The SSI-T model generally predicts the binary experimental data accurately with respect to the transition pressures and the overall total adsorbed amount across the range of pressure and composition. The prediction for the 50% mixture of the two sorbates deviates from the experimental data both with respect to the maximum capacity and the transition pressure—features that were also noted by the authors in their original analysis. The SSI-T model predictions of the individual species are also shown in Figure 8b (dotted and dashed lines) and are used to compute the competitive selectivity of the strongly adsorbing C_3_H_8_ over C_2_H_6_, $S_{C_{3}H_{8}}$ (shown as black line in the figure). The latter is computed as^64^

17 $$S_{C_{3}H_{8}}=\frac{{\left\langle N\right\rangle }_{C_{3}H_{8}}^{m}}{{\left\langle N\right\rangle }_{C_{2}H_{6}}^{m}}\frac{1-y_{C_{3}H_{8}}}{y_{C_{3}H_{8}}}$$

where ${\left\langle N\right\rangle }_{C_{8}H_{8}}^{m}$ and ${\left\langle N\right\rangle }_{C_{2}H_{6}}^{m}$ are the molar adsorbed amounts of the two species at equilibrium, and $y_{C_{3}H_{8}}$ is the molar bulk phase composition. The competitive selectivity for the binary mixtures is greatest as *p* → 0 (i.e., Henry selectivity) and decreases with pressure up to the point at which the phase transition occurs. Here, the competitive selectivity increases sharply before decreasing again monotonically after complete transition. For rigid adsorbents that typically exhibit linear or concave physisorption isotherms with respect to pressure, the competitive selectivity would typically decrease monotonically with pressure, resulting in a trade-off between equilibrium selectivity (and thus product purity) and working capacity. The selectivity behavior outlined by COMOC-2 points to the potential of using this adsorbent in an adsorption-based process (e.g., pressure swing adsorption) to separate this mixture, ideally operating at an inlet pressure corresponding to the maximum competitive selectivity for the feed composition, and extracting the product at a pressure below the phase-transition pressure for pure C_8_H_8_ to maximize the purity of the recovered product and the working capacity (total amount of product recovered) for the separation. While this is promising, more detailed process modeling that accounts for heat and mass transfer dynamics will be necessary to assess the true separation performance—a task that can be carried out using the competitive SSI-T model and its parametrization presented above.

#### 3.2.2 Binary Adsorption of Water and Carbon Dioxide in CALF-20

CALF-20 is a metal organic framework that exhibits high selectivity toward CO_2_ over N_2_, retains high CO_2_ adsorption capacity in the presence of moderate amounts of water vapor, can be synthesized sustainably at scale, and is robust toward steam and high temperature.^62^ These properties make CALF-20 a promising candidate for postcombustion carbon dioxide capture from humid gas streams, as highlighted by its excellent cyclic stability and process performance in lab-scale demonstrations.^65^ The retention of CO_2_ capacity in the presence of water, in particular, is one of the most important features of CALF-20, enabling process cycles that operate with a lower energy penalty in a capture process compared to hydrophilic benchmark materials such as Zeolite 13X.^66^ From a thermodynamic and mechanistic perspective, adsorption in CALF-20 exhibits some unique properties that are at the origin of its unique performance.

The experimental unary equilibrium adsorption isotherms for H_2_O and CO_2_ in CALF-20 at various temperatures obtained from Lin et al.^62^ and Nguyen et al.^61^ are shown in Figure 9a as black and red symbols. Note that the data were measured on a structured form of CALF-20, consisting of 80% MOF and 20% polysulfone binder. Assuming an inert binder, the data in Figure 9a were obtained by converting the reported isotherms from a mass basis of structured material to that of the active MOF. We note that the shapes of the equilibrium isotherms differ considerably for the two species, with CO_2_ showing Type I isotherms characteristic of other CO_2_ capture adsorbents such as Zeolite 13X, and H_2_O displaying sigmoidal isotherms with respect to pressure. The sigmoidal shape of the H_2_O isotherms indicates some form of an adsorption-induced transformation to the configuration of adsorbed molecules, leading to a stronger affinity of the host framework to adsorbed H_2_O molecules at higher bulk concentrations, while such a transition is not induced by the presence of CO_2_ molecules at equilibrium.^67^

**Figure 9:**
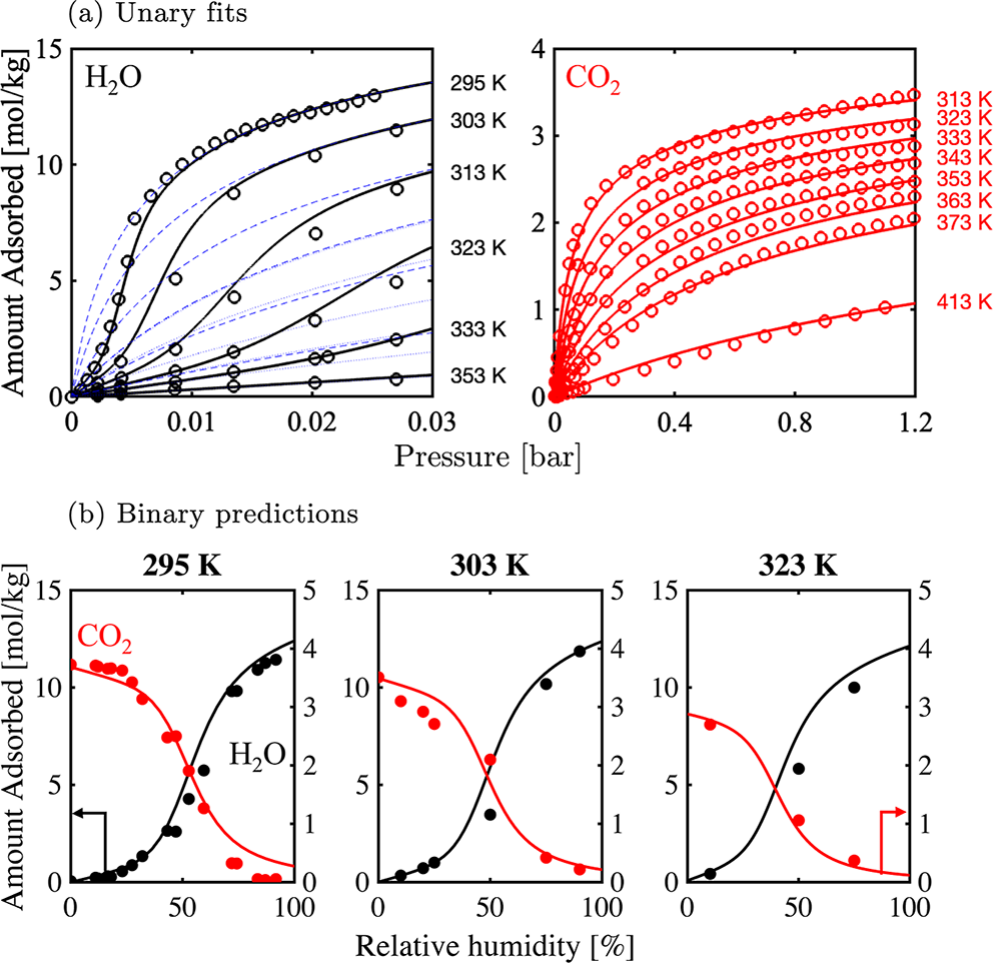
(a) Absolute unary adsorption isotherms of H_2_O (black), and CO_2_ on CALF-20 as a function of pressure at various temperatures (labeled in the outset to the right of each panel). The empty symbols correspond to equilibrium data reported by Lin et al.^62^ and Nguyen et al.,^61^ and the solid lines represent the SSI-T model parametrization given by eq 11 and the parameters in Table 4. The equilibrium data for H_2_O were first used to fully parametrize the SSI-T model to obtain the corresponding sorbate-dependent parameters for H_2_O and the sorbate-independent parameters for CALF-20. CALF-20 interacts with CO_2_ in the same manner in both phases and therefore exhibits the same parameters. (b) Binary adsorption equilibrium for H_2_O (black), and CO_2_ (red) in CALF-20 at different temperatures as a function of relative humidity. The filled symbols correspond to competitive adsorption equilibrium data reported by Lin et al.^62^ and Constant et al.,^60^ and the solid lines correspond to SSI-T model predictions computed using eq 14.

The SSI-T model was applied to describe the data such that CALF-20 undergoes a structural transition between two phases with equal void volume but different Henry’s law constants for H_2_O (i.e., $K_{0}$ and −Δ*u*_ads_), while it conserves the same properties in both phases for CO_2_ (equivalent to the original SSI formulation). The resulting model fits are shown in Figure 9a as black and red solid lines and the hypothetical SSI isotherms for H_2_O in each phase as blue dashed lines. The corresponding model parameters are given in Table 4. Similar to the previously modeled systems, the SSI-T model provides an excellent representation of the unary equilibrium data across a wide range of temperatures for both sorbates. The model parameters estimated indicate a simple configurational transition for the adsorbed water molecules (i.e., a change in the configurational partition function for an adsorbed molecule *Z*(1)) represented by a change in the Henry’s law parameters $K_{0}$ and −Δ*u*_ads_, without any structural changes to the CALF-20 framework. This transition is reversible and triggered when the free energy difference between the two states, *F*_0_^host^(*v*_uc_,*T*) = *U*_0_^host^-*TS*_0_^host^, is overcome by the thermodynamic potential of the adsorbed phase. The state of the adsorbed CO_2_ molecules is unaffected by this transition, and thus the adsorption of CO_2_ is described by the same parameters in both phases.

**Table 4:** SSI-T Model Parameters Estimated to Describe the Adsorption and Desorption Equilibria of H_2_O and CO_2_ on CALF-20

CALF-20		
**Parameter**	**H**_**2**_ **O**	**CO**_**2**_
	**Phase I**	
$K_{0}^{I}$ [molec. cavity^–1^ bar^–1^]	1.05 × 10^–5^	1.78 × 10^–5^
-Δ*u*_ads_^I^[kJ mol^–1^]	43.50	40.08
*b* ^I^ [Å^3^/molec.]	16.8	77.5
	**Phase II**	
$K_{0}^{II}$ [molec. cavity^–1^ bar^–1^]	3.44 × 10^–6^	$$K_{0}^{I}$$
-Δ*u*_ads_^II^[kJ mol^–1^]	50.66	-Δ*u*_ads_^I^
*b* ^II^ [Å^3^/molec.]	*b* ^I^	
	**Structural parameters**	
*v* ^I^ [Å^3^/cavity]	380	
*v* ^II^ [Å^3^/cavity]	*v* ^I^	
Δ*U*_0_^host^[kJ mol^–1^]	24.96	
Δ*S*_0_^host^[J mol^–1^ K^–1^]	26.57	
ΔΩ^stress^ [kJ mol^–1^]	0	

The experimental binary H_2_O/CO_2_ adsorption equilibrium data from Lin et al.^62^ and Constant et al.^60^ are shown as filled black and red symbols in Figure 9b. The experimental data set shows a favorable retention of CO_2_ adsorption capacity in CALF-20 up to approximately 40% relative humidity across the range of temperatures, with better retention at lower temperatures. Using the unary parametrization of the SSI-T model for this system, we predict the binary adsorption equilibria at these conditions and show the results as solid lines in Figure 9b. The SSI-T provides an accurate prediction of the competitive adsorption behavior in this complex system, correctly predicting the suppression of water adsorption (and the step in the water isotherm) in the presence of CO_2_ and correspondingly the retention of CO_2_ capacity as observed in binary experimental data.

#### 3.2.3 Binary Adsorption of Carbon Dioxide and Methane in MIL-53­(Al)

We return to MIL-53­(Al) to predict the binary adsorption of CO_2_ and CH_4_ and validate the predictions with data reported by Ortiz et al.,^63^ using the unary parametrizations of the system described in the previous section. Figure 10 shows experimental isotherms at 273 K measured at 2.75, 3.5, and 4 bar for various compositions of the two gases as filled symbols. In this data set, Ortiz et al. reported total amounts adsorbed and desorbed for both species, and so, the SSI-T model predictions of the two species were combined to obtain the corresponding prediction. The SSI-T curves for the adsorption and desorption branches for this system are shown as red and blue solid lines, respectively, with the phase diagram for this system as a function of pressure and CO_2_ mole fraction at 273 K shown to the right of the isotherms. Once again, using the unary parametrization, the SSI-T model accurately predicts the equilibrium behavior in both the adsorption and desorption branches. The model also correctly predicts the phase transition behavior across a range of pressure and CO_2_ mole fraction as seen from the phase diagram, where the experimental phase boundaries are shown as green solid lines. The accuracy of the prediction for this system, given its complexity arising from the presence of multiple transitions and a relatively low free energy barrier, deteriorates under certain conditions. For such complex systems, deterioration can be countered by using some binary equilibrium data within the parameter estimation to supplement the unary data that can be used exclusively to parametrize most systems.

**Figure 10:**
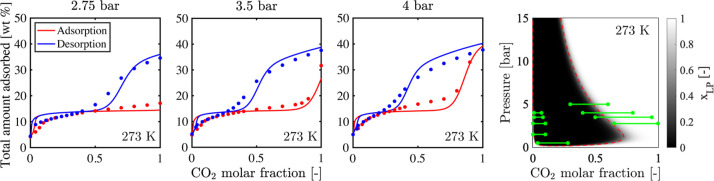
Binary adsorption (red) and desorption (blue) equilibrium for CO_2_ and CH_4_ in MIL-53­(Al) at 273 K as a function of CO_2_ composition at different total pressures. The filled symbols correspond to the total adsorbed amount of the two sorbates in weight percentage reported by Ortiz et al.^63^ and the solid lines correspond to SSI-T model predictions computed using eq 14. The panel on the right shows the phase diagram for this system as a function of pressure and CO_2_ mole fraction at 273 K, with horizontal green lines to show the experimentally observed coexistence region.

### 3.3 Accounting for Unequal-Sized Molecules and Comparison with the Langmuir-STA Model

The SSI-T model carries many similarities in structure to the STA model proposed by Hiraide et al.^26^ The latter is also based on the description of the adsorbent as a surface consisting of discrete domains that can exist in two distinct phases. However, a key assumption of the STA model is that each phase is described as a simple Langmuir surface, i.e., these domains each contain a finite number of sites (*S* ^I^ and *S* ^II^) where the guest molecules can interact with any of the free adsorption sites on any of the domains, i.e., (*M* – *m*)*S* ^I^ and *mS* ^II^ for the two phases. The STA model is given as

18 $$\begin{array}{ll} {\left\langle N\right\rangle }^{STA,m} & =\left(1-x\right)\frac{q_{I}^{s,STA}K^{I}\left(T\right)p}{1+{\;K}^{I}\left(T\right)p}+x\frac{q_{II}^{s,STA}K^{II}\left(T\right)p}{1+{\;K}^{II}\left(T\right)p}\\ x & =\frac{{\left[\frac{{\left(1+K^{II}\left(T\right)p\right)}^{q_{II}^{s,STA}}}{{\left(1+K^{I}\left(T\right)p\right)}^{q_{I}^{s,STA}}}\exp\left(\frac{\Delta F_{host}}{RT}\right)\right]}^{s}}{1+{\left[\frac{{\left(1+K^{II}\left(T\right)p\right)}^{q_{II}^{s,STA}}}{{\left(1+K^{I}\left(T\right)p\right)}^{q_{I}^{s,STA}}}\exp\left(\frac{\Delta F_{host}}{RT}\right)\right]}^{s}} \end{array}$$

where Δ*F*_host_ = Δ*U*_host_ – *T*Δ*S*_host_ + ΔΩ^stress^ is the free-energy barrier for a phase transition [J mol^–1^], *q*_*X*_^s,STA^ is the Langmuir saturation capacity [mol kg^–1^] for phase *X*, and *s* is a sorbate-independent constant that relates to the size of the domain that behaves as a Langmuir surface. The difference with our formulation is that in the SSI-T model, a free molecule in the bulk interacts with a cavity of volume *v* and maximum capacity ⌊*v*/*b*⌋ as a whole, rather than a single site within the cavity. This assumption allows the host in a given phase to occlude different numbers of guest molecules depending on their molecular size, which is not possible if each phase is assumed to behave as a thermodynamically consistent Langmuir surface.

Figure 11 compares the SSI-T model (solid lines) with the STA model (dashed lines) for the adsorption and desorption of C_2_H_6_, and C_3_H_8_ on COMOC-2. These two sorbates occupy significantly different adsorbed volumes (48.2 and 73.1 Å^3^/molec. respectively, as given in Table 3), and so would exhibit proportionally different molar saturation capacities per unit mass or volume of adsorbent, as correctly predicted by the SSI-T model. For a Langmuir model to be thermodynamically consistent, however, the adsorbent is required to have the same number of *sites* in each phase. As a result, the STA model underpredicts the adsorption of C_2_H_6_. As such, the model is limited to the description of isotherms for a single sorbate or multiple sorbates with similar saturation capacities.

**Figure 11:**
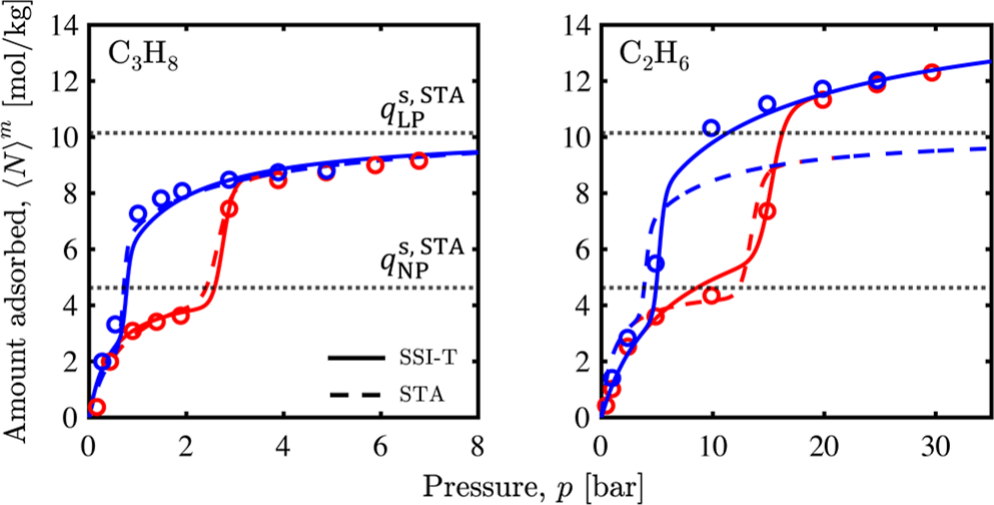
Comparison of SSI-T (solid) and thermodynamically consistent STA isotherm (dashed) model fits to absolute unary adsorption (red) and desorption (blue) isotherms of C_2_H_6_, and C_3_H_8_ on COMOC-2 as a function of pressure at 303 K on COMOC-2.^59^ The horizontal dotted lines correspond to fixed saturation capacities (*q*_NP_^s,STA^ and *q*_LP_^s,STA^) for the two phases obtained by fitting the STA model to C_3_H_8_ isotherms. The sorbate–dependent parameters for C_2_H_6_ ( $K^{I}{\left(T\right)}_{{0,C}_{2}H_{6}}$ and $\Delta u_{{ads,C}_{2}H_{6}}^{I}$ ) were then fitted by fixing the sorbate-independent parameters Δ*U*_host_, Δ*S*_host_, ΔΩ^stress^, and *s*.

### 3.4 Influence of Effective Domain Size on Observed Transition Behavior

The concept of effective domain in the SSI-T model was introduced in the methods section, defined as a collection of η unit cells that can independently undergo a phase transition and illustrated schematically in Figure 12c for η = 2, 4, and 12. In the SSI-T model, this concept is used as a practical means to model interactions (i.e., cooperative and restrictive) between neighboring unit cells. As a consequence, the effective domain size has a direct impact on the macroscopic phase transition behavior for the same adsorbent, as illustrated by the adsorption equilibrium isotherms shown in Figure 12a. A larger effective domain (i.e., a greater range of cooperation between unit cells) corresponds to a greater proportion of the adsorbent transitioning independently at a given ΔΩ^os^ = 0, leading to a sharper step in the isotherm. Conversely, a smaller domain (with a lower bound of the unit cell) corresponds to a wider distribution of coexisting phases within the adsorbent and thus a more gradual transition with increasing *p*.

**Figure 12:**
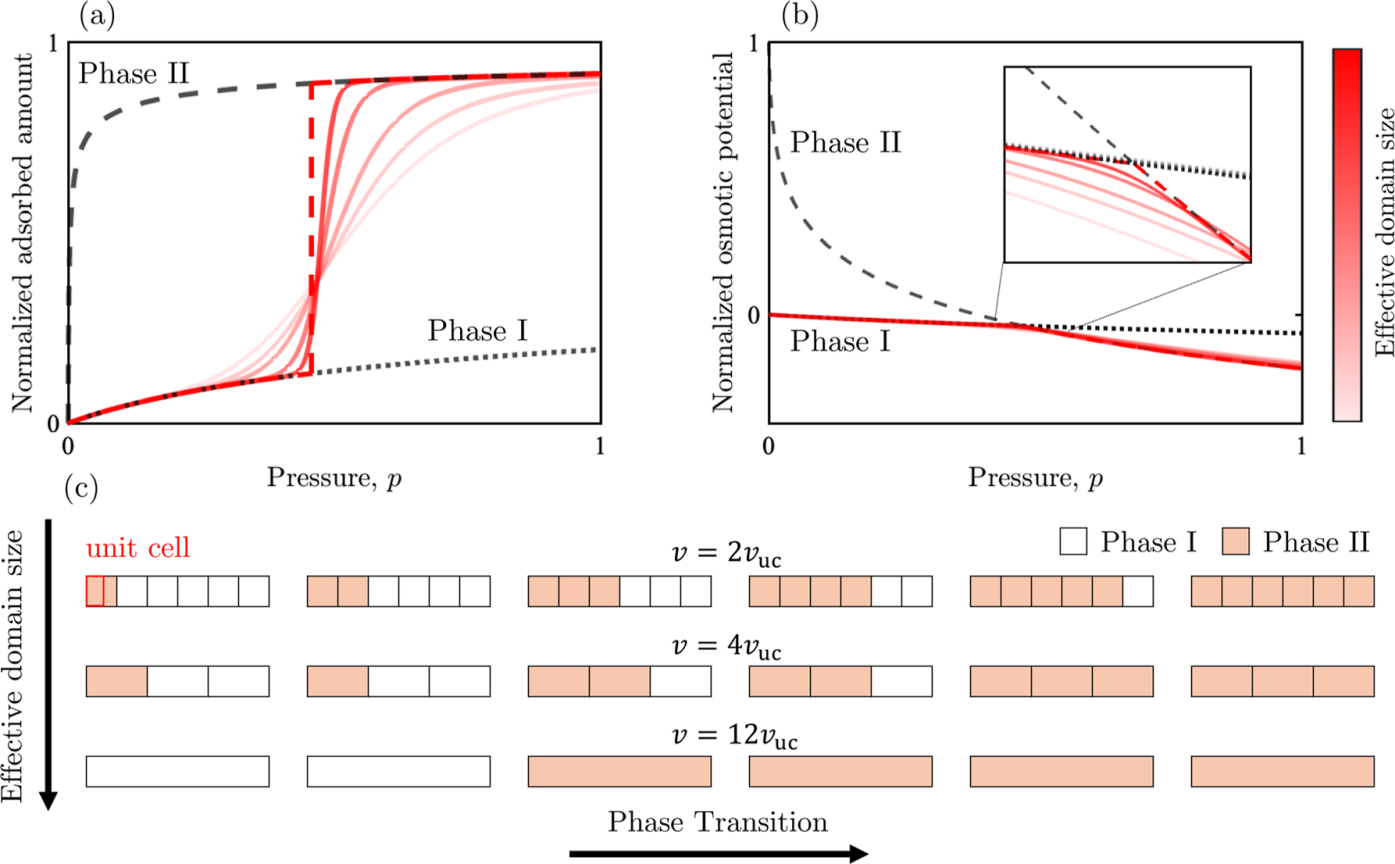
Conceptual illustration of the impact of effective domain size on the macroscopically observed phase-transition behavior of a representative flexible adsorbent using the SSI-T model. (a) Macroscopically observed equilibrium isotherms for the same adsorbent, normalized by its maximum saturation capacity, for a range of hypothetical cases where the effective domain is of different sizes. A larger effective domain leads to a sharper and earlier transition, while a smaller domain leads to a shallower transition, with the descriptions of the two hypothetical phases being essentially identical at the macroscopic scale. The dashed red line corresponds to a case where the number of unit cells per domain tends to ∞. (b) Normalized osmotic potential for the same flexible adsorbent and the different domain sizes computed at the same temperature (red lines) and the two hypothetical phases I and II (dotted and dashed black lines, respectively) as a function of pressure. The vertical axis is normalized by the free energy difference of the empty host (i.e., *p* → 0) between the two phases. The system with a larger effective domain exhibits a higher osmotic potential at the transition pressure (see inset) compared to the systems with smaller domains. (c) Schematic illustration of the concept of the effective domain, showing how a smaller domain can represent a finer resolution independent of transitions within the larger adsorbent, and so lead to a more gradual isotherm to be observed and a complete transition occurring at a higher pressure.

Figure 12b shows the corresponding osmotic free energy profiles computed using the SSI-T model as a function of pressure. For a larger effective domain, the free energy barrier is overcome abruptly as the osmotic potential of the framework increases, leading to a sudden transition, with the complete transition occurring at a much lower pressure. This follows from Second Law thermodynamics that a smaller domain size (i.e., larger number of subsystems) is energetically favored over a larger domain, due to possessing greater entropy. Conversely, in the thermodynamic limit (η → ∞ and *v* ≫ *b*), the void volume of the effective domain approaches the total volume of all voids in the adsorbent (i.e., the micropore volume), and the entire adsorbent will transition simultaneously, resulting in a discontinuous isotherm. As such, while a domain consisting of a single unit cell is the most energetically favorable for isolated unit cells, a larger effective domain is often observed to satisfy physical constraints such as shared structural elements (e.g., rotating ligands, interconnected layers, etc.), and propagation of transition-induced strain across unit cells in a larger crystallite.

We hypothesize that the ability to relate the parameter η to morphological properties of the flexible adsorbent at the pore and crystal scales can be exploited to inform the tailored design and synthesis of adsorbents with specific structural properties. Conversely, the SSI-T model itself can be integrated within process simulators to carry out inverse-design of flexible adsorbents to identify desired material properties (e.g., shape and width of the phase-coexistence region through Δ*F*_host_ and the effective domain size, and single phase properties through K(T) ) for different adsorption-based applications. In addition to the ordered crystalline flexible adsorbents evaluated in this work, the proposed approach could also be directly applied to describe adsorption-induced transitions in disordered materials, such as polymers and carbons, for applications in gas storage, separations, and other sustainable energy-related applications.

### 3.5 State Dependence of Isosteric Enthalpy of Adsorption and Net-Enthalpy of Phase-Transition

The isosteric enthalpy of adsorption, Δ*h* ^ads^, can be defined as the heat released per unit fluctuation in adsorbed amount from a given state of equilibrium. This is given by the derivative of ln *p* with respect to 1/*kT* at constant loading as follows

19 $$\Delta h^{ads}\left(p,T\right)=-{\frac{\partial \ln p}{\partial \left(1/kT\right)}|}_{\left\langle N\right\rangle }=-\frac{\left|\Delta u_{ads}^{I}\right|A+\left|\Delta u_{ads}^{II}\right|B+q_{net}C}{A+B+C}$$

where

20 $$A=\left(1-x_{\theta }\right){\frac{\partial {\left\langle N\right\rangle }^{I}}{\partial \ln p}|}_{\left(1/kT\right)}$$

21 $$B=x_{\theta }{\frac{\partial {\left\langle N\right\rangle }^{II}}{\partial \ln p}|}_{\left(1/kT\right)}$$

22 $$C=x_{\theta }\left(1-x_{\theta }\right){\left[{\left\langle N\right\rangle }^{II}-{\left\langle N\right\rangle }^{I}\right]}^{2},,X\in \left\{I,II\right\}$$

The derivation of eq 19 and the explicit expressions for *A*, *B*, and *C* are given in the Supporting Information. The variables *A* and *B* represent the sensitivity of the adsorbed amount to pressure within fixed phases I and II adjusted by their respective phase fractions, while *C* describes the susceptibility to transition between the two phases due to adsorption or desorption. In other words, *A* and *B* describe cases where adsorption of an additional molecule does not lead to a phase transition, whereas *C* accounts for cases where adsorption of an additional molecule results in a transition. As such, the sum of these three variables corresponds to the total observable variance in occupancy (i.e., observed adsorbed amount ⟨N⟩ ) with pressure. Accordingly, the terms $\left|\Delta u_{ads}^{I}\right|A$ and $\left|\Delta u_{ads}^{II}\right|B$ describe the variance in adsorption energy within a fixed phase where, $\left|\Delta u_{ads}^{I}\right|$ and $\left|\Delta u_{ads}^{II}\right|$ are the energy change per additional adsorbed molecule in each phase. The final contribution *q*_net_ *C* describes the energetic fluctuations from switching from one phase to another, where *q*_net_ describes the energy change per additional adsorbed molecule that leads to a phase transition. This is given by

23 $$q_{net}=\frac{{\left\langle N\right\rangle }^{II}\left|\Delta u_{ads}^{II}\right|-{\left\langle N\right\rangle }^{I}\left|\Delta u_{ads}^{I}\right|-\Delta U_{0}^{host}-\Delta \Omega^{stress}}{{\left\langle N\right\rangle }^{II}-{\left\langle N\right\rangle }^{I}}$$

Note that for the limits *x*_θ_ = 0 and *x*_θ_ = 1, eq 19 reduces to $-\left|\Delta u_{ads}^{I}\right|$ and $-\left|\Delta u_{ads}^{II}\right|$ respectively, i.e., the solution for a single-phase system. For intermediate values (i.e., phase-coexistence region), the total enthalpy change includes the contribution from *q*_net_, which manifests as a suppression of the exothermic enthalpy of adsorption by the endothermic enthalpy of phase transition.^68^ We also note that the result in eq 19 is identical in structure to that reported for the Langmuir-based STA model reported by Hiraide et al. for *n* = *l* = 1, i.e., when the saturation capacities for each of the two phases are equal to one molecule per domain. This proof is provided in the Supporting Information.

Figure 13 shows −Δ*h* ^ads^ as a function of adsorbed amount calculated for the adsorption and desorption of CO_2_, C_2_H_6_, N_2_, CH_4_, and Ar on ZIF-7 at two different temperatures. The results show the characteristic trough of the isosteric enthalpy of adsorption that is triggered by the phase transition in flexible adsorbents and that contributes substantially to reducing the exothermic enthalpy of adsorption. Notably, Δ*h* ^ads^ is dependent on temperature within the phase-coexistence region for all five sorbates, and independent of temperature and adsorbed amount when a single phase is present. This is expected as the phase transition is triggered at lower adsorbed amounts with increasing temperature, as seen in Figure 6, and the contribution to the overall change in enthalpy from the enthalpy of phase transition (and thus *q*_net_) is only available when the adsorption of an additional molecule leads to a change in the phase fraction *x*_θ_. Along the same lines, Δ*h* ^ads^ is also dependent on the direction of transition (i.e., adsorption or desorption, depending on the sign of ΔΩ^stress^), which is shown by the apparent hysteresis between red and blue curves. We highlight two features of this hysteresis loop: as with the temperature dependence, the NP to LP phase transition is triggered at a higher adsorbed amount than that at which the reverse LP to NP transition is completed on the desorption branch. Second, the extent of the maximum reduction in Δ*h* ^ads^ within the phase-coexistence region is greater for adsorption than for desorption. This is due to the larger endothermic contribution to *q*_net_ during adsorption due to the positive ΔΩ^stress^, as opposed to being negative for desorption. This property offers flexible adsorbents with unique intrinsic thermal management properties, which may be beneficial in certain applications. From a process modeling perspective, it is vital to correctly model Δ*h* ^ads^ as a variable that is both state and path-dependent (e.g., by using eq 22) to avoid overpredicting the heat generated during adsorption, which can consequently yield incorrect predictions of process-scale performance.

**Figure 13:**
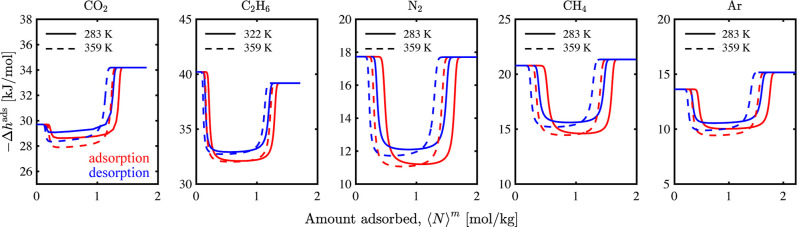
Isosteric enthalpy of adsorption computed using eq 19 for the adsorption (red) and desorption (blue) isotherms of CO_2_, C_2_H_6_, N_2_, CH_4_, and Ar on ZIF-7 as a function of adsorbed amount. The solid and dashed lines correspond to calculations for the highest and lowest temperatures, respectively, at which isotherm data were measured by Yang et al.^28^ and shown in Figure 6.

## 4 Conclusions

In this work, we have presented a novel approach to modeling the adsorption and desorption equilibria in flexible adsorbents. To our knowledge, the resulting simplified statistical isotherm for transition materials (SSI-T) is the first continuous and differentiable explicit isotherm model that accurately describes experimental unary adsorption equilibrium data and correctly predicts binary equilibrium for the range of systems tested. The model is parametrized for reversible and irreversible (hysteretic) systems with ten or nine adjustable parameters, respectively, which consist of sorbate-dependent and independent parameters. The five (or four for reversible systems) sorbate-independent parameters are constant for a given flexible adsorbent and so can be fixed for estimating the isotherm parameters for different sorbates for the same material. We have validated the model against experimental equilibrium data for four different flexible adsorbents that display a range of guest-induced transitions, namely, gate-opening, breathing, and configurational transitions—showing the versatility of this model in describing the continuous transitions and predicting the conditions for phase transition across a range of adsorbates. We demonstrated the excellent predictive capabilities of the model when used to describe competitive binary adsorption and desorption equilibria following its parametrization on the corresponding unary equilibrium data. We have also applied the SSI-T model to quantify the state-dependence of the isosteric enthalpy of adsorption, particularly the influence of the endothermic phase-transition on the net enthalpy change during the process of adsorption and adsorption-induced phase transition. The latter offers flexible adsorbents with unique intrinsic thermal management properties in adsorption-based applications such as separations. These unique features underscore the potential of the SSI-T model for use in numerical adsorption process simulators, which require the ability to model the equilibrium at a wide range of conditions.

## Supplementary Material



## Notes

(1-2) additional details of the SSI-T model derivation, (3) details on modeling hysteresis using the SSI-T model, (4) list of model parameters and their bounds for parameter estimation, (5) SSI-T model parametrization and predictions for CO_2_/H_2_O adsorption in CAU-10H and Al-Fumarate, and (6) derivation of the isosteric enthalpy of adsorption for the SSI-T model (PDF)
