# Supplementary material for: Generalized Statistical Isotherm for Modeling Adsorption Equilibria in Stimuli-Responsive Framework Materials
Source: Langmuir. 2026 Jun 17;42(25):17924–42. doi: 10.1021/acs.langmuir.6c01192 (PMC13325879; doi:10.1021/acs.langmuir.6c01192)
Supplement: Supplementary file 1 [file la6c01192_si_001.pdf]

Supporting Information for

Generalized Statistical Isotherm for Modeling Adsorption Equilibria in Stimuli–Responsive Framework Materials

Hassan Azzan,[†,*] David Danaci,[†,‡,¶] Camille Petit,[†] Ronny Pini[†]

Contents

S1 Adsorption in a *frozen* framework: Simplified Statistical Isotherm **2**

S2 Derivation of the Grand Partition Function for the SSI-T Isotherm **6**

S3 Modeling Full and Partial Reversal Curves within the Major Hysteresis Loop **7**

S4 Parameter Estimation: Model Parameter Bounds and Types **9**

S5 Unary Parameterization and Binary Predictions for CO_2/H_2O adsorption in CAU-10H and Al-Fumarate **10**

S6 Derivation of Isosteric Enthalpy of Adsorption for the SSI-T model **12**

[†] Department of Chemical Engineering, Imperial College London, London, SW7 2AZ, United Kingdom
[‡] The Sargent Centre for Process Systems Engineering, Imperial College London, London, SW7 2AZ, United Kingdom
[¶] I-X Centre for AI in Science, Imperial College London, London, W12 0BZ, United Kingdom

[*] Corresponding author: hassan.azzan15@imperial.ac.uk

S1 Adsorption in a *frozen* framework: Simplified Statistical Isotherm

In this section, we give a summary of the derivation of the SSI model to highlight the key assumptions that were used in the development of our model. This model applies to any adsorbent that can be described as a *frozen solid* (*i.e.*, solid with a fixed void volume) for which we extend the definition to include a fixed adsorption energy and Henry law constant for a given adsorbate.

The adsorbent is assumed to be in a reservoir at the macroscopic limit such that the total volume of the reservoir and the number of adsorbate molecules in the reservoir tend to infinity. The adsorbent, in thermal and chemical equilibrium (temperature T and chemical potential μ) with the reservoir, consists of M identical and non-interacting cavities of fixed volume v with a maximum occupancy of ω adsorbate molecules under a potential field $U(r)$. Under these assumptions, the system is described by the Grand canonical ensemble (μVT ensemble), for which the canonical partition function $\mathcal{Q}$ can be written in terms of the activity of the adsorbate a as follows.

$$\mathcal{Q} = \left(\sum_{i=0}^{\omega} Z(i) a^i \right)^M \equiv \mathcal{Z}^M \tag{S1}$$

Here, $Z(i)$ is the configurational partition function for i adsorbed molecules in a cavity, and is given by the integral of the total potential energy $U(r_1, \ldots, r_i)$ of the cavity containing i adsorbed molecules over their coordinates r_i,

$$Z(i) = \frac{1}{i!} \int_v \exp\left[\frac{-U(r_1, \ldots, r_i)}{kT} \right] \, \mathrm{d}r_1 \cdots \, \mathrm{d}r_i \tag{S2}$$

and $\mathcal{Z}$ is the canonical partition function for a single cavity. Since $\mathcal{Q}$ is defined by the product of the partition functions of the M cavities. Applying the assumption of identical and non-interacting cavities, this is equivalent to $\mathcal{Z}$ raised to the power of M. The ensemble average number of adsorbed particles per cavity $\langle N \rangle$ is thus obtained by differentiating the grand potential for each cavity ($\ln \mathcal{Z}$) with respect to activity.

$$\langle N \rangle = a \left(\frac{\partial \ln \mathcal{Z}}{\partial a} \right) = \frac{\sum_{i=1}^{\omega} i Z(i) a^i}{\sum_{i=0}^{\omega} Z(i) a^i} \tag{S3}$$

Equation S3 is the general definition of an adsorption isotherm using the grand canonical ensemble consisting of identical and non-interacting *sites.* As an obvious example, at the limit of $\omega = 1$, the potential field $U(r)$ becomes uniform, and the system can be solved analytically, yielding the Langmuir isotherm[1]. However, for many systems with more than one adsorbed molecule, it is not possible to obtain a closed-form expression for $Z(i)$ and needs to be approximated based on suitable assumptions. Ruthven[2] provided a method for such an approximation by making the following simplifying assumptions.

1. The gas phase is assumed to be ideal, and the activity of the bulk phase as a function of the pressure p [Pa] and temperature T [K] is given by $a = p/kT$,

2. Molecules in neighboring cavities do not interact, and each cavity can be regarded as independent with a void volume v,

3. The interaction between guest molecules (hard spheres of an effective molecular volume of b) is described by the Sutherland potential[3] with negligible attractive interaction between adsorbed molecules,

4. The adsorption potential within a cavity is uniform and the interaction between a single guest molecule and the host is described by Henry's law (*i.e.*, $\lim_{a\to 0} \langle N \rangle = \mathcal{K}(T)a$, where $\mathcal{K}(T)$ is the temperature-dependent Henry's law constant given in terms of a pre-exponent K_0 and adsorption energy $-\Delta u$ by $\mathcal{K}(T) = K_0 \exp\left[-\Delta u_{\text{ads}}/kT\right]$), and

5. The maximum number of adsorbed molecules per cavity ω is determined by a reduction of free volume within the cavity (*i.e.*, floor of the ratio of cavity and effective molecular volumes, $\omega = \lfloor v/b \rfloor$).

These assumptions are applied to factorize Equation S2 into three independent contributions that can be computed analytically. First, the potential field experienced by adsorbed molecules is described by the Sutherland potential for hard spheres of diameter σ as follows.

$$U(r) = -\epsilon \left(\frac{\sigma}{r}\right)^6, \qquad r \geq \sigma \tag{S4}$$

This provides two of the three contributions to $Z(i)$; $Z_{\text{rep}}(i)$ to describe the repulsive forces due to free volume constraints, and $Z_{\text{att}}(i)$, which is the mean-field contribution describing attractive forces between adsorbed molecules.

$$Z_{\text{rep}}(i) = \frac{1}{i!}\left(1 - ib/v\right)^i \tag{S5}$$

$$Z_{\text{att}}(i) = \exp\left[\frac{ib\epsilon}{vkT}\right] \tag{S6}$$

Next, the interaction between the adsorbent and each molecule is assumed to contribute equally to $Z(i)$ and is therefore given by the interaction of a single molecule with the potential field ($Z(1)$). This is given by Henry's law (described by the temperature-dependent constant $\mathcal{K}(T) = K_0 \exp\left[-\Delta u/kT\right]$) as follows[4].

$$Z(1)^i = \left(\frac{\mathcal{K}(T)kT}{1 - b/v}\right)^i \tag{S7}$$

The pre-exponential factor K_0 and the internal energy change due to adsorption Δu can be estimated by matching the model to experimental isotherms measured at different temperatures. Finally, the product of these three contributions yields Ruthven's closed-form factorization for $Z(i)$,

$$Z(i) = Z(1)^i Z_{\text{rep}}(i) Z_{\text{att}}(i) = \frac{(\mathcal{K}(T)kT)^i}{i!}\left(\frac{1 - ib/v}{1 - b/v}\right)^i \exp\left[\frac{ib\epsilon}{vkT}\right] \tag{S8}$$

and combining Equations S3 and S8 gives the simplified statistical isotherm $\langle N \rangle^{\text{SSI}}$ for a single adsorbing component ideal gas (*i.e.*, $a = p/kT$) is given by the following.

$$\langle N \rangle^{\text{SSI}} = \frac{\mathcal{K}(T)p + \sum_{i=2}^{\omega} \frac{(\mathcal{K}(T)p)^i}{(i-1)!}\left(\frac{1-ib/v}{1-b/v}\right)^i \exp\left[\frac{ib\epsilon}{vkT}\right]}{1 + \mathcal{K}(T)p + \sum_{i=2}^{\omega} \frac{(\mathcal{K}(T)p)^i}{i!}\left(\frac{1-ib/v}{1-b/v}\right)^i \exp\left[\frac{ib\epsilon}{vkT}\right]}, \tag{S9}$$

$$i \geq 2 \tag{S10}$$

At the limit of b approaching v, the SSI reduces to the Langmuir isotherm. As discussed above, the SSI describes the ensemble average number of adsorbed molecules per cavity [molec. cavity^{-1}] and needs to be converted to a mass basis $\langle N \rangle^{\text{SSI,m}}$ [mol kg^{-1}] to model experimental data. Several methods for this conversion have been described in the literature. For instance, isotherms resulting from molecular dynamics simulations or other crystallographic assessments are typically defined using units of molecules per unit cell (volume of void plus the volume of solid). For such cases, the conversion is done by calculating the number of unit cells per unit mass using the molar mass of a unit cell and the framework density obtained from crystallographic data[5]. However, since the SSI is modeled per cavity (only the void volume) we carry out this conversion by assuming that the average volume of a single cavity is v [m^3] and the sum of the total volume of these cavities per unit mass is equal to the micropore volume v_{mic} [m^3 kg^{-1}]. This unit conversion can be given as follows.

$$\langle N \rangle^{\text{SSI,m}} = \frac{\langle N \rangle^{\text{SSI}}}{N_A} \frac{v_{\text{mic}}}{v} \tag{S11}$$

For many cases, the contribution to $Z(i)$ from intermolecular attractions $Z_{\text{att}}(i)$ can be neglected as the magnitude of ϵ is typically much smaller than the Δu describing the Henry's law constant. Regardless, unless both are explicitly defined, the two energetic contributions can be combined into a single parameter Δu for fitting purposes. Thus, the model is fully defined using 4 parameters, v, b, K_α^0, and Δu. For rigid frameworks, the empty volume of a cavity v (independent of sorbate) is obtained directly from crystallographic data and is given by ϕv_{uc} where ϕ and v_{uc} are the unit cell volume and void fraction. This approximation is a valid approximation for rigid frameworks with non-interacting cavities. The micropore volume can be readily obtained experimentally using sub-critical nitrogen or argon sorption experiments, and the remaining 3 sorbate-dependent parameters can be fitted to match experimental isotherms.

The extension of this model to predict binary and ternary adsorption equilibria has been reported in subsequent publications[6–9]. In brief, for a system consisting of two adsorbing species α and β, the grand partition function for a single cavity $\mathcal{Z}^{\alpha\beta}$ is computed by summing over all possible combinations of i and j that satisfy $ib + jb_\beta \leq v$ for the two components respectively (once again neglecting intermolecular attractions, *i.e.*, $\epsilon \ll \Delta u$) as

$$\mathcal{Z}^{\alpha\beta} = \sum_{j=0} \sum_{i=0} Z(i,j) a_\alpha^i a_\beta^j \tag{S12}$$

where,

$$Z(i,j) = \frac{Z(1,0)^i Z(0,1)^j}{i!j!} \left(1 - ib_\alpha/v - jb_\beta/v\right)^{i+j} \tag{S13}$$

Here, the free volume reduction term $Z_{\text{rep}}(i)$ is modified to include the second component, and $Z(1,0)$ and $Z(0,1)$ are equivalent to $Z(1)$ (given by Equation S7) for species α and β respectively. Accordingly, the competitive adsorption of component α in a binary system at partial pressures p_α and p_β is given by

$$\langle N \rangle_\alpha^{\text{SSI}}(p_\alpha, p_\beta, T) = \frac{\mathcal{K}_\alpha(T)p_\alpha + \sum_j \sum_i \frac{(\mathcal{K}_\alpha(T)p_\alpha)^i (\mathcal{K}_\beta(T)p_\beta)^j}{(i-1)!j!} \frac{\left(1 - ib_\alpha/v - jb_\beta/v\right)^i}{(1-b_\alpha/v)^i (1-b_\beta/v)^j}}{1 + \mathcal{K}_\alpha(T)p_\alpha + \mathcal{K}_\beta(T)p_\beta + \sum_j \sum_i \frac{(\mathcal{K}_\alpha(T)p_\alpha)^i (\mathcal{K}_\beta(T)p_\beta)^j}{i!j!} \frac{\left(1 - ib_\alpha/v - jb_\beta/v\right)^i}{(1-b_\alpha/v)^i (1-b_\beta/v)^j}}, \quad i+j \geq 2 \tag{S14}$$

where the summations are carried out for all values of $i + j \geq 2$ and $ib_\alpha + jb_\beta \leq v$. When $b_\alpha + b_\beta$ is greater than, but the two are individually smaller than v, the binary SSI isotherm reduces to the extended Langmuir isotherm where two molecules of different species compete for a single adsorption site. The two-sorbate system using the SSI model is fully defined using 7 fitted parameters.

S2 Derivation of the Grand Partition Function for the SSI-T Isotherm

The probability that m out of M cavities exist in phase II is given by the following.

$$\begin{aligned}\rho_\theta^{\text{host}}(m) &\propto \binom{M}{m} \exp\left[(M-m)\frac{-F_{\text{I}}^{\text{host}}(T)}{kT}\right] \exp\left[m\frac{-F_{\text{II}}^{\text{host}}(T)}{kT}\right] \\ &\propto \binom{M}{m}\left(\exp\left[\frac{-\Delta F_\theta^{\text{host}}(T)}{kT}\right]\right)^m \end{aligned} \tag{S15}$$

The grand partition function $\mathcal{Q}^{\text{trans}}$ is given by the sum of the partition functions of all possible states of a system, which for the above system is given as,

$$\begin{aligned}\mathcal{Q}^{\text{trans}} &= \sum_{m=0}^{M} \rho_\theta^{\text{host}}(m)[\mathcal{Z}^{\text{I}}]^{M-m}[\mathcal{Z}^{\text{II}}]^m \\ &= \sum_{m=0}^{M}\binom{M}{m}\left(\exp\left[\frac{-\Delta F_\theta^{\text{host}}(T)}{kT}\right]\right)^m \left(\sum_{n=0}^{\omega^{\text{I}}} Z^{\text{I}}(n)a^n\right)^{M-m}\left(\sum_{l=0}^{\omega^{\text{II}}} Z^{\text{II}}(l)a^l\right)^m \\ &= \left[\left(\sum_{n=0}^{\omega^{\text{I}}} Z^{\text{I}}(n)a^n\right) + \exp\left[\frac{-\Delta F_\theta^{\text{host}}(T)}{kT}\right]\left(\sum_{l=0}^{\omega^{\text{II}}} Z^{\text{II}}(l)a^l\right)\right]^M \equiv (\mathcal{Z}^{\text{trans}})^M \end{aligned} \tag{S16}$$

and, given that the ensemble average adsorbed amount assuming an ideal gas phase is given by $\langle N\rangle(p,T) = \frac{p}{\mathcal{Z}}\frac{\partial \mathcal{Z}}{\partial p}$, the SSI-T isotherm $\langle N\rangle_\theta^{\text{SSI-T}}(p,T)$ is given by

$$\langle N\rangle_\theta^{\text{SSI-T}}(p,T) = \frac{\left[\sum_{n=1}^{\omega^{\text{I}}}\frac{(\mathcal{K}^{\text{I}}(T)p)^n}{(n-1)!}\left(\frac{1-nb/v^{\text{I}}}{1-b/v^{\text{I}}}\right)^n\right] + \exp\left[\frac{-\Delta F_\theta^{\text{host}}(T)}{kT}\right]\left[\sum_{l=1}^{\omega^{\text{II}}}\frac{(\mathcal{K}^{\text{II}}(T)p)^l}{(l-1)!}\left(\frac{1-lb/v^{\text{II}}}{1-b/v^{\text{II}}}\right)^l\right]}{\left[\sum_{n=0}^{\omega^{\text{I}}}\frac{(\mathcal{K}^{\text{I}}(T)p)^n}{(n)!}\left(\frac{1-nb/v^{\text{I}}}{1-b/v^{\text{I}}}\right)^n\right] + \exp\left[\frac{-\Delta F_\theta^{\text{host}}(T)}{kT}\right]\left[\sum_{l=0}^{\omega^{\text{II}}}\frac{(\mathcal{K}^{\text{II}}(T)p)^l}{(l)!}\left(\frac{1-lb/v^{\text{II}}}{1-b/v^{\text{II}}}\right)^l\right]} \tag{S17}$$

Given that $\langle N\rangle^{\text{X}} = \frac{\left[\sum_{n=1}^{\omega^{\text{X}}}\frac{(\mathcal{K}^{\text{X}}(T)p)^n}{(n-1)!}\left(\frac{1-nb/v^{\text{X}}}{1-b/v^{\text{X}}}\right)^n\right]}{\mathcal{Z}^{\text{X}}}$ for any phase X, we can make the following substitutions

$$\langle N\rangle_\theta^{\text{SSI-T}}(p,T) = \frac{\langle N\rangle^{\text{I}}\mathcal{Z}^{\text{I}} + \exp\left[\frac{-\Delta F_\theta^{\text{host}}(T)}{kT}\right]\langle N\rangle^{\text{II}}\mathcal{Z}^{\text{II}}}{\mathcal{Z}^{\text{I}} + \exp\left[\frac{-\Delta F_\theta^{\text{host}}(T)}{kT}\right]\mathcal{Z}^{\text{II}}} \tag{S18}$$

Dividing both the numerator and denominator by $\mathcal{Z}^{\text{I}}$ and rearranging by collecting the coefficients for $\langle N\rangle^{\text{X}}$, we get the SSI-T isotherm in terms of the variable phase fraction x_θ.

$$\begin{aligned}\langle N\rangle_\theta^{\text{SSI-T}}(p,T) &= \frac{1}{1+\frac{\mathcal{Z}^{\text{II}}}{\mathcal{Z}^{\text{I}}}\exp\left[\frac{-\Delta F_\theta^{\text{host}}(T)}{kT}\right]}\langle N\rangle^{\text{I}} + \frac{\frac{\mathcal{Z}^{\text{II}}}{\mathcal{Z}^{\text{I}}}\exp\left[\frac{-\Delta F_\theta^{\text{host}}(T)}{kT}\right]}{1+\frac{\mathcal{Z}^{\text{II}}}{\mathcal{Z}^{\text{I}}}\exp\left[\frac{-\Delta F_\theta^{\text{host}}(T)}{kT}\right]}\langle N\rangle^{\text{II}} \\ \langle N\rangle_\theta^{\text{SSI-T}}(p,T) &= (1-x_\theta)\langle N\rangle^{\text{I}} + x_\theta\langle N\rangle^{\text{II}}\end{aligned} \tag{S19}$$

S3 Modeling Full and Partial Reversal Curves within the Major Hysteresis Loop

For this derivation, we require a mathematical definition for the *direction* of transition. From a given initial phase composition of x_0, the direction is defined by the change in the ratio between the canonical partition functions for the adsorbed molecules in the two phases ($\mathcal{Z}^{\mathrm{II}}/\mathcal{Z}^{\mathrm{I}}$) – *e.g.* for a single adsorbate, increasing pressure would would cause a positive change in this ratio, and vice versa.

Following from the assumption of non-interacting cavities, the path of lowest free energy is obtained by scaling the major branch (solid lines in Figure 4(a)) with the ratio of the initial fraction of cavities in phase II at the start of the switch x_0 (a constant) to the phase fraction along the major loop in the same direction ($x_{\mathrm{ads}}(p,T)$ and $x_{\mathrm{des}}(p,T)$ – solid lines in Figure 4(b)). For cases where the switch is from adsorption to desorption, this is given by

$$\langle N \rangle^{\text{SSI-T}}_{\Delta\mathcal{Z}<0}(p,T) = \left(1 - \frac{x_0}{x_{\mathrm{ads}}(p,T)}\right) \langle N \rangle^{\text{SSI-T}}_{\mathrm{des}}(p,T) + \frac{x_0}{x_{\mathrm{ads}}(p,T)} \langle N \rangle^{\text{SSI-T}}_{\mathrm{ads}}(p,T) \tag{S20}$$

and for the reverse by the following.

$$\langle N \rangle^{\text{SSI-T}}_{\Delta\mathcal{Z}>0}(p,T) = \left(1 - \frac{x_0}{x_{\mathrm{des}}(p,T)}\right) \langle N \rangle^{\text{SSI-T}}_{\mathrm{ads}}(p,T) + \frac{x_0}{x_{\mathrm{des}}(p,T)} \langle N \rangle^{\text{SSI-T}}_{\mathrm{des}}(p,T) \tag{S21}$$

This feature of the model does not require any additional parameters, as the minor loops are fully defined using the major loop parameterization. The resulting complete reversal curves (*i.e.*, partial to full transition) and corresponding phase composition as a function of pressure are illustrated in Figure 4 as blue and red dot-dashed lines. This same formulation can also be used to model partial reversal curves (one state of partial transition to another at a different pressure) within the hysteresis envelope, This same formulation can also be used to model partial reversal curves (one state of partial transition

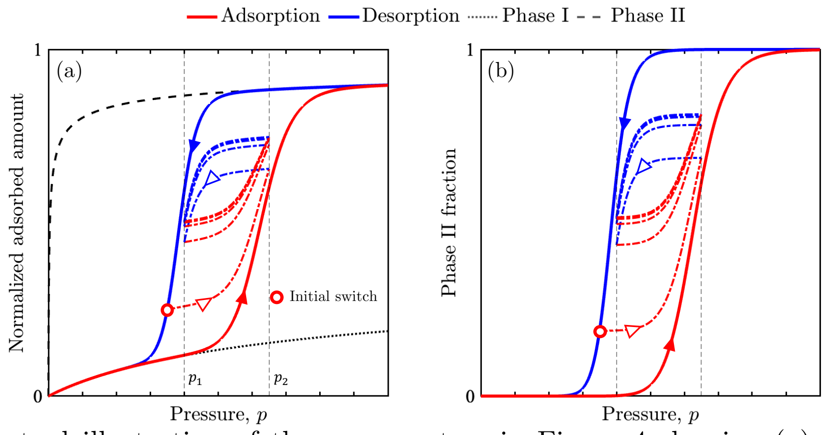

Figure S1: Conceptual illustration of the same system in Figure 4 showing (a) the adsorption and desorption isotherms in the major and minor loops. Here, the minor loops correspond to partial reversal curves where the pressure is switched between two points within the major hysteresis loop, and (b) the corresponding phase diagram for the same isotherms showing the mole fraction of Phase II present in the adsorbent as a function of pressure for the major and minor loops.

to another at a different pressure) within the hysteresis envelope. For such cases, the partial reversal curves form loops when the system is cycled between two states within the hysteresis region, as shown in Figure S1. For this system, the pressure is switched on the desorption branch of the major loop, following which the system is cycled between two fixed pressures, p_1 and p_2. Upon repeated cycling,

the system approaches a closed loop within the phase envelope. The model equation presented is an explicit function of state variables pressure and temperature, and the direction of the transition given by the change in the ratio between the canonical partition functions for the adsorbed molecules in the two phases ($\mathcal{Z}^{\mathrm{II}}/\mathcal{Z}^{\mathrm{I}}$), and can be easily implemented using a simple algorithm.

This approach has been used extensively to model history-dependent hysteresis and reversal curves in ferromagnetism[10], and falls into the category of scaling models, where the resulting reversal curve is a scaled summation of the complete reversal curves. Takakura *et al.*[11] reported a simplified scaling method to model hysteresis in flexible adsorbents that can be used in process-scale evaluations. However, the scaling method used in their work is only valid for a restricted number of systems, such as ELM-11, that present flat horizontal isotherms above and below the transition conditions, as the reversal curve is scaled by a constant value (*i.e.*, the saturation capacity of the large pore phase). Such an approach would not work as a general method for all flexible adsorbents as the scaling would result in the partial reversal curves crossing the major reversal curves under certain conditions, *e.g.* when the isotherms for two phases are monotonically increasing above and below the transition.

S4 Parameter Estimation: Model Parameter Bounds and Types

Table S1: Parameter bounds and variable types used in the parameter estimation routine, described in the parameter estimation section of the Main Manuscript.

Parameter	Unit	Bounds		Variable Type
		Low	High	
**Sorbate Dependent Parameters**				
$\mathcal{K}_0^{\mathrm{I}}$	molec. cavity^{-1} bar^{-1}	10^{-7}	1	Continuous
$\mathcal{K}_0^{\mathrm{II}}$	molec. cavity^{-1} bar^{-1}	10^{-7}	1	Continuous
b^{I}	$\mathrm{\AA}^3$/molec	10	140	Continuous
b^{II}	$\mathrm{\AA}^3$/molec	10	140	Continuous
$-\Delta u_{\mathrm{ads}}^{\mathrm{I}}$	kJ mol^{-1}	0	120	Continuous
$-\Delta u_{\mathrm{ads}}^{\mathrm{II}}$	kJ mol^{-1}	0	120	Continuous
**Sorbate Independent/Structural Parameters**				
v^{II}	$\mathrm{\AA}^3$/cavity	100	6000	Continuous
$v^{\mathrm{I}}/v^{\mathrm{II}}$	-	0	1	Continuous
$\Delta U_0^{\mathrm{host}}$	kJ mol^{-1}	0	120	Continuous
$\Delta S_0^{\mathrm{host}}$	J mol^{-1} K^{-1}	0	500	Continuous
$\Delta \Omega^{\mathrm{stress}}$	kJ mol^{-1}	0	10	Continuous

S5 Unary Parameterization and Binary Predictions for CO_2/H_2O adsorption in CAU-10H and Al-Fumarate

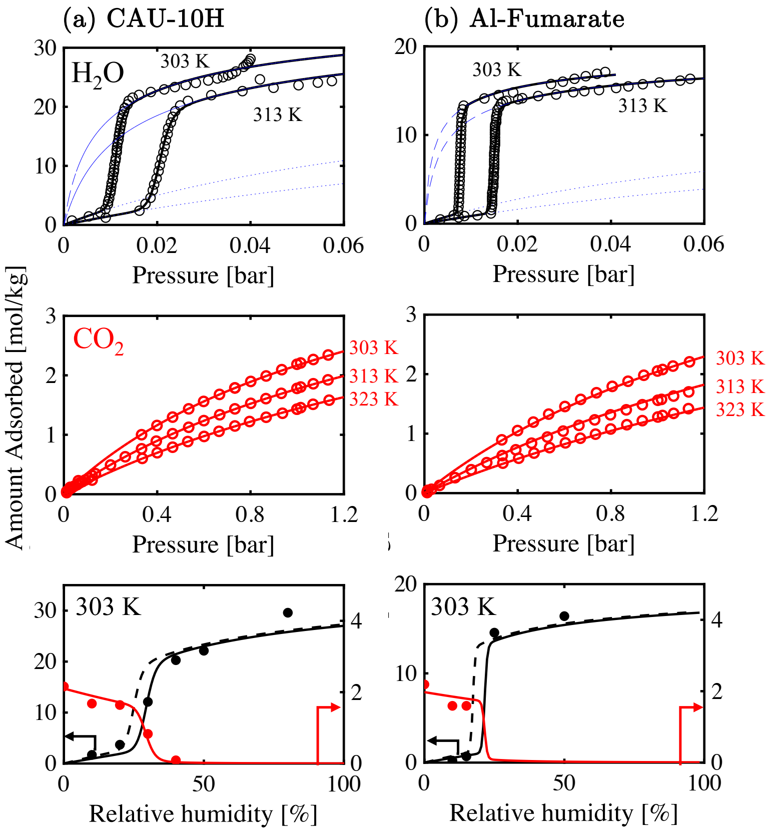

Figure S2: Absolute unary and binary adsorption isotherms of H_2O (black) and CO_2 on (a) CAU-10H and (b) Al-Fumarate as a function of pressure at various temperatures (labeled in the outset to the right of each panel). The empty symbols correspond to equilibrium data reported by Constant *et al.*[12], and the solid lines represent the SSI-T model parameterization given by Equation 11 and the parameters in Tables S2 and S3. The equilibrium data for H_2O were first used to fully parameterize the SSI-T model to obtain the corresponding sorbate-dependent parameters for H_2O and the sorbate-independent parameters.

Table S2: The SSI-T model parameters estimated to parameterize the adsorption and desorption equilibria of H_2O and CO_2 on CAU10-H.

CAU10-H

Parameter	H_2O	CO_2
	**Phase I**	
$\mathcal{K}_0^{I}$ [molec. cavity^{-1} bar^{-1}]	2.38×10^{-4}	2.11×10^{-4}
$-\Delta u_{ads}^{I}$ [kJ mol^{-1}]	47.66	27.65
b^{I} [Å^3/molec]	18.63	42.56
	**Phase II**	
$\mathcal{K}_0^{II}$ [molec. cavity^{-1} bar^{-1}]	1.35×10^{-6}	$\mathcal{K}_0^{I}$
$-\Delta u_{ads}^{II}$ [kJ mol^{-1}]	50.51	$-\Delta u_{ads}^{I}$
b^{II} [Å^3/molec]	b^{I}	
	**Structural parameters**	
v^{I} [Å^3/cavity]	1810	
v^{II} [Å^3/cavity]	v^{I}	
ΔU_0^{host} [kJ mol^{-1}]	231.51	
ΔS_0^{host} [J mol^{-1} K^{-1}]	1579.90	
$\Delta \Omega^{stress}$ [kJ mol^{-1}]	0	

Table S3: The SSI-T model parameters estimated to parameterize the adsorption and desorption equilibria of H_2O and CO_2 on Al-Fumarate.

Al-Fumarate

Parameter	H_2O	CO_2
	**Phase I**	
$\mathcal{K}_0^{I}$ [molec. cavity^{-1} bar^{-1}]	2.38×10^{-4}	1.62×10^{-4}
$-\Delta u_{ads}^{I}$ [kJ mol^{-1}]	49.28	25.53
b^{I} [Å^3/molec]	14.06	42.56
	**Phase II**	
$\mathcal{K}_0^{II}$ [molec. cavity^{-1} bar^{-1}]	2.98×10^{-7}	$\mathcal{K}_0^{I}$
$-\Delta u_{ads}^{II}$ [kJ mol^{-1}]	52.51	$-\Delta u_{ads}^{I}$
b^{II} [Å^3/cavity]	b^{I}	
	**Structural parameters**	
v^{I} [Å^3/cavity]	725	
v^{II} [Å^3/cavity]	v^{I}	
ΔU_0^{host} [kJ mol^{-1}]	8.11	
ΔS_0^{host} [J mol^{-1} K^{-1}]	269.74	
$\Delta \Omega^{stress}$ [kJ mol^{-1}]	0	

S6 Derivation of Isosteric Enthalpy of Adsorption for the SSI-T model

The SSI-T isotherm $\langle N\rangle^{\text{SSI-T}}$ as a function of pressure p and temperature T is given as,

$$\langle N\rangle_\theta^{\text{SSI-T}} = (1-x_\theta)\,\langle N\rangle^{\text{I}} + x_\theta\,\langle N\rangle^{\text{II}} \tag{S22}$$

where N_{X} gives the SSI isotherm for each of the two essentially rigid phases as,

$$\langle N\rangle^{\text{X}} = \frac{\sum_{i=1}^{\omega^{\text{X}}} \frac{(\mathcal{K}^{\text{X}}(T)p)^i}{(i-1)!}\left(\frac{1-ib/v_{\text{X}}}{1-b/v_{\text{X}}}\right)^i}{\sum_{i=0}^{\omega^{\text{X}}} \frac{(\mathcal{K}^{\text{X}}(T)p)^i}{i!}\left(\frac{1-ib/v_{\text{X}}}{1-b/v_{\text{X}}}\right)^i}, \qquad i \geq 2, \qquad X \in \{I, II\} \tag{S23}$$

and, x_θ is the phase fraction (with respect to phase II) given as below.

$$x_\theta = \frac{\dfrac{\sum_{l=0}^{\omega^{\text{II}}} \frac{(\mathcal{K}^{\text{II}}(T)p)^l}{l!}\left(\frac{1-lb/v^{\text{II}}}{1-b/v^{\text{II}}}\right)}{\sum_{n=0}^{\omega^{\text{I}}} \frac{(\mathcal{K}^{\text{I}}(T)p)^n}{n!}\left(\frac{1-nb/v^{\text{I}}}{1-b/v^{\text{I}}}\right)} \exp\left[\dfrac{-\Delta F_\theta^{\text{host}}(T)}{kT}\right]}{1+\dfrac{\sum_{l=0}^{\omega^{\text{II}}} \frac{(\mathcal{K}^{\text{II}}(T)p)^l}{l!}\left(\frac{1-lb/v^{\text{II}}}{1-b/v^{\text{II}}}\right)}{\sum_{n=0}^{\omega^{\text{I}}} \frac{(\mathcal{K}^{\text{I}}(T)p)^n}{n!}\left(\frac{1-nb/v^{\text{I}}}{1-b/v^{\text{I}}}\right)} \exp\left[\dfrac{-\Delta F_\theta^{\text{host}}(T)}{kT}\right]} = \frac{\frac{\mathcal{Z}^{\text{II}}}{\mathcal{Z}^{\text{I}}} \exp\left[\dfrac{-\Delta F_\theta^{\text{host}}(T)}{kT}\right]}{1+\frac{\mathcal{Z}^{\text{II}}}{\mathcal{Z}^{\text{I}}} \exp\left[\dfrac{-\Delta F_\theta^{\text{host}}(T)}{kT}\right]} = \frac{y}{1+y} \tag{S24}$$

The isosteric enthalpy of adsorption Δh^{ads} for the SSI-T model is computed using the Clausius-Clapeyron equation given as,

$$\Delta h^{\text{ads}} = -\left.\frac{\partial \ln p}{\partial(1/kT)}\right|_{\langle N\rangle^{\text{SSI-T}}} = -\frac{\left.\frac{\partial\langle N\rangle^{\text{SSI-T}}}{\partial(1/kT)}\right|_{\ln p}}{\left.\frac{\partial\langle N\rangle^{\text{SSI-T}}}{\partial \ln p}\right|_{1/kT}} \tag{S25}$$

From the above, the partial derivatives of $\langle N\rangle^{\text{SSI-T}}$ with respect of $\ln p$ and $1/kT$ are required to obtain an analytical expression for Δh^{ads}. Applying the product rule, the two derivatives are given as follows.

$$\left.\frac{\partial\langle N\rangle^{\text{SSI-T}}}{\partial \ln p}\right|_{(1/kT)} = (1-x_\theta)\left.\frac{\partial\langle N\rangle^{\text{I}}}{\partial \ln p}\right|_{(1/kT)} + x_\theta\left.\frac{\partial\langle N\rangle^{\text{II}}}{\partial \ln p}\right|_{(1/kT)} + \left.\frac{\partial x_\theta}{\partial \ln p}\right|_{(1/kT)}\left[\langle N\rangle^{\text{II}} - \langle N\rangle^{\text{I}}\right] \tag{S26}$$

$$\left.\frac{\partial\langle N\rangle^{\text{SSI-T}}}{\partial(1/kT)}\right|_{\ln p} = (1-x_\theta)\left.\frac{\partial\langle N\rangle^{\text{I}}}{\partial(1/kT)}\right|_{\ln p} + x_\theta\left.\frac{\partial\langle N\rangle^{\text{II}}}{\partial(1/kT)}\right|_{\ln p} + \left.\frac{\partial x_\theta}{\partial(1/kT)}\right|_{\ln p}\left[\langle N\rangle^{\text{II}} - \langle N\rangle^{\text{I}}\right] \tag{S27}$$

For phase X, the derivative of the SSI equation with respect to $\ln p$ using the quotient rule is given as follows.

$$\left.\frac{\partial \langle N\rangle^{\mathrm{X}}}{\partial \ln p}\right|_{(1/kT)} = \frac{\sum_{i=1}^{\omega^{\mathrm{X}}} \frac{(\mathcal{K}^{\mathrm{X}}(T)p)^i}{(i-2)!}\left(\frac{1-ib/v_{\mathrm{X}}}{1-b/v_{\mathrm{X}}}\right)^i}{\sum_{i=0}^{\omega^{\mathrm{X}}} \frac{(\mathcal{K}^{\mathrm{X}}(T)p)^i}{i!}\left(\frac{1-ib/v_{\mathrm{X}}}{1-b/v_{\mathrm{X}}}\right)^i} + \left[\frac{\sum_{i=1}^{\omega^{\mathrm{X}}} \frac{(\mathcal{K}^{\mathrm{X}}(T)p)^i}{(i-1)!}\left(\frac{1-ib/v_{\mathrm{X}}}{1-b/v_{\mathrm{X}}}\right)^i}{\sum_{i=0}^{\omega^{\mathrm{X}}} \frac{(\mathcal{K}^{\mathrm{X}}(T)p)^i}{i!}\left(\frac{1-ib/v_{\mathrm{X}}}{1-b/v_{\mathrm{X}}}\right)^i}\right]^2$$

$$\equiv \langle N\rangle^{\mathrm{X}} - [\langle N\rangle^{\mathrm{X}}]^2 + \frac{\sum_{i=1}^{\omega^{\mathrm{X}}}(i-1)\frac{(\mathcal{K}^{\mathrm{X}}(T)p)^i}{(i-1)!}\left(\frac{1-ib/v_{\mathrm{X}}}{1-b/v_{\mathrm{X}}}\right)^i}{\sum_{i=0}^{\omega^{\mathrm{X}}} \frac{(\mathcal{K}^{\mathrm{X}}(T)p)^i}{i!}\left(\frac{1-ib/v_{\mathrm{X}}}{1-b/v_{\mathrm{X}}}\right)^i} \tag{S28}$$

$$A = (1-x_\theta)\left.\frac{\partial \langle N\rangle^{\mathrm{I}}}{\partial \ln p}\right|_{(1/kT)}, \qquad B = x_\theta \left.\frac{\partial \langle N\rangle^{\mathrm{II}}}{\partial \ln p}\right|_{(1/kT)}. \tag{S29}$$

Next, recalling that $\left.\frac{\partial \ln \mathcal{Z}^{\mathrm{X}}}{\partial \ln p}\right|_{1/kT} = \langle N\rangle^{\mathrm{X}}$ and $x_\theta = \frac{y}{1+y}$, the derivative of x_θ with respect to $\ln p$ is obtained as follows.

$$\frac{\partial x_\theta}{\partial \ln p} = \frac{\partial x_\theta}{\partial \ln y}\frac{\partial \ln y}{\partial \ln p} = \frac{y}{(1+y)^2}\left[\frac{\partial \ln \mathcal{Z}^{\mathrm{II}}}{\partial \ln p} - \frac{\partial \ln \mathcal{Z}^{\mathrm{I}}}{\partial \ln p}\right] \equiv x_\theta(1-x_\theta)\left[\langle N\rangle^{\mathrm{II}} - \langle N\rangle^{\mathrm{I}}\right] \tag{S30}$$

$$C = \frac{\partial x_\theta}{\partial \ln p}\left[\langle N\rangle^{\mathrm{II}} - \langle N\rangle^{\mathrm{I}}\right] = x_\theta(1-x_\theta)\left[\langle N\rangle^{\mathrm{II}} - \langle N\rangle^{\mathrm{I}}\right]^2 \tag{S31}$$

Similarly, we compute the derivatives of $\langle N\rangle^{\mathrm{X}}$ with respect to $1/kT$ at constant $\ln p$, by applying a change of variable $\phi = K^{\mathrm{X}}(T)p$ and the chain rule.

$$\left.\frac{\partial \langle N\rangle^{\mathrm{X}}}{\partial(1/kT)}\right|_{\ln p} = \left.\frac{\partial \langle N\rangle^{\mathrm{X}}}{\partial \ln \phi}\frac{\partial \ln \phi}{\partial(1/kT)}\right|_{\ln p} \tag{S32}$$

Given the definition of the Henry's law constant $K^{\mathrm{X}}(T) = K^0_{\mathrm{X}} \exp\left(\frac{|\Delta u^{\mathrm{X}}_{\mathrm{ads}}|}{kT}\right)$, and $\phi \propto p$, the two derivatives can be written as follows.

$$\frac{\partial \langle N\rangle^{\mathrm{X}}}{\partial \ln \phi} = \left.\frac{\partial \langle N\rangle^{\mathrm{X}}}{\partial \ln p}\right|_{(1/kT)} \tag{S33}$$

$$\frac{\partial \ln \phi}{\partial(1/kT)} = |\Delta u^{\mathrm{X}}_{\mathrm{ads}}| \tag{S34}$$

Substituting Equation S26 into S34, we obtain an expression for $\left.\frac{\partial \langle N\rangle^{\mathrm{X}}}{\partial(1/kT)}\right|_{\ln p}$.

$$\left.\frac{\partial \langle N\rangle^{\mathrm{X}}}{\partial(1/kT)}\right|_{\ln p} = |\Delta u^{\mathrm{X}}_{\mathrm{ads}}| \left.\frac{\partial \langle N\rangle^{\mathrm{X}}}{\partial \ln p}\right|_{(1/kT)} \tag{S35}$$

The derivative of the phase fraction x_θ with respect to $1/kT$ is given by,

$$\frac{\partial x_\theta}{\partial(1/kT)} = \frac{\partial x_\theta}{\partial \ln y}\frac{\partial \ln y}{\partial(1/kT)} \tag{S36}$$

where the derivative of y with respect to $1/kT$ needs to be derived, recalling that $\Delta F_\theta^{\text{host}}(T) = \Delta U_0^{\text{host}} - T\Delta S_0^{\text{host}} + \Delta\Omega^{\text{stress}}$ is a function of temperature. This is given as follows.

$$\begin{aligned}\frac{\partial \ln y}{\partial(1/kT)} &= |\Delta u_{\text{ads}}^{\text{II}}|\frac{\partial \ln \mathcal{Z}^{\text{II}}}{\partial \ln p} - |\Delta u_{\text{ads}}^{\text{I}}|\frac{\partial \ln \mathcal{Z}^{\text{I}}}{\partial \ln p} - \Delta U_0^{\text{host}} - \Delta\Omega^{\text{stress}} \\ &\equiv |\Delta u_{\text{ads}}^{\text{II}}| \langle N\rangle^{\text{II}} - |\Delta u_{\text{ads}}^{\text{I}}| \langle N\rangle^{\text{I}} - \Delta U_0^{\text{host}} - \Delta\Omega^{\text{stress}} \end{aligned} \tag{S37}$$

Combining, Equation S37 with S36, and using the definition for $\frac{\partial x_\theta}{\partial \ln y}$ from Equation S30, we obtain the derivative of x_θ with respect to $1/kT$ as,

$$\frac{\partial x_\theta}{\partial(1/kT)} = x_\theta(1-x_\theta)\left[|\Delta u_{\text{ads}}^{\text{II}}| \langle N\rangle^{\text{II}} - |\Delta u_{\text{ads}}^{\text{I}}| \langle N\rangle^{\text{I}} - \Delta U_0^{\text{host}} - \Delta\Omega^{\text{stress}}\right] \tag{S38}$$

$$= x_\theta(1-x_\theta)q_{\text{net}}\left[\langle N\rangle^{\text{II}} - \langle N\rangle^{\text{I}}\right] \tag{S39}$$

where, q_{net} is the net enthalpy of adsorption corresponding to a case where the adsorption of one molecule leads to a phase transition and is given as follows.

$$q_{\text{net}} = \frac{|\Delta u_{\text{ads}}^{\text{II}}| \langle N\rangle^{\text{II}} - |\Delta u_{\text{ads}}^{\text{I}}| \langle N\rangle^{\text{I}} - \Delta U_0^{\text{host}} - \Delta\Omega^{\text{stress}}}{\langle N\rangle^{\text{II}} - \langle N\rangle^{\text{I}}} \tag{S40}$$

Finally, plugging in these results into Equation S26 and Equation S27, and taking the ratio, we obtain the analytical expression for Δh^{ads} using the SSI-T model.

$$\Delta h^{\text{ads}} = -\left.\frac{\partial \ln p}{\partial(1/kT)}\right|_{\langle N\rangle^{\text{SSI-T}}} = -\frac{|\Delta u_{\text{ads}}^{\text{I}}|A + |\Delta u_{\text{ads}}^{\text{II}}|B + q_{\text{net}}C}{A+B+C} \tag{S41}$$

Taking the limit of $n = 1$ and $l = 1$, corresponding to a case where each phase can occlude exactly 1 molecule, we get the Langmuir case $\langle N\rangle^{\text{X}} = \frac{\mathcal{K}^{\text{X}}(T)p}{1+\mathcal{K}^{\text{X}}(T)p}$ for the SSI-T model given by, the variables A, B, and C reduces to

$$A = (1-x_\theta)\left[\langle N\rangle^{\text{I}} - [\langle N\rangle^{\text{I}}]^2\right] = \frac{\mathcal{K}^{\text{I}}(T)p}{(1+\mathcal{K}^{\text{I}}(T)p)^2} \tag{S42}$$

$$B = x_\theta\left[\langle N\rangle^{\text{II}} - [\langle N\rangle^{\text{II}}]^2\right] = \frac{\mathcal{K}^{\text{II}}(T)p}{(1+\mathcal{K}^{\text{II}}(T)p)^2} \tag{S43}$$

$$C = x_\theta(1-x_\theta)[\langle N\rangle^{\text{II}} - \langle N\rangle^{\text{I}}]^2 \tag{S44}$$

which is the exact result obtained by Hiraide *et al.* for the STA model assuming a single molecule per domain in each phase.

References

[1] Langmuir, I. The adsorption of gases on plane surfaces of glass, mica and platinum. *J. Am. Chem. Soc.* **1918**, *40*, 1361–1403.

[2] Ruthven, D. M. Simple Theoretical Adsorption Isotherm for Zeolites. *Nat. Phys. Sci.* **1971**, *232*, 70–71.

[3] Hill, T. L. *An introduction to statistical thermodynamics*; Dover Publications: New York, 1986 - 1960.

[4] Ruthven, D. M. A simple theoretical isotherm for zeolites: further comments. *Zeolites* **1982**, *2*, 242–243.

[5] Boutin, A.; Coudert, F.-X.; Springuel-Huet, M.-A.; Neimark, A. V.; Férey, G.; Fuchs, A. H. The Behavior of Flexible MIL-53(Al) upon CH_4 and CO_2 Adsorption. *J. Phys. Chem. C* **2010**, *114*, 22237–22244.

[6] Ruthven, D.; Loughlin, K.; Holborow, K. Multicomponent sorption equilibrium in molecular sieve zeolites. *Chem. Eng. Sci.* **1973**, *28*, 701–709.

[7] Miller, G. W.; Knaebel, K. S.; Ikels, K. G. Equilibria of nitrogen, oxygen, argon, and air in molecular sieve 5A. *AIChE J.* **1987**, *33*, 194–201.

[8] Wedler, C.; Ferre, A.; Azzan, H.; Danaci, D.; Petit, C.; Pini, R. Binary Adsorption Equilibria of Three CO_2+CH_4 Mixtures on NIST Reference Zeolite Y (RM 8850) at Temperatures from 298 to 353 K and Pressures up to 3 MPa. *J. Chem. Eng. Data* **2024**, *69*, 4216–4229.

[9] Kaminsky, R. D.; Monson, P. A. An analysis of the statistical model adsorption isotherm. *AIChE J.* **1992**, *38*, 1979–1989.

[10] Mörée, G.; Leijon, M. Review of Hysteresis Models for Magnetic Materials. *Energies* **2023**, *16*, 3908.

[11] Takakura, Y.; Sugimoto, S.; Fujiki, J.; Kajiro, H.; Yajima, T.; Kawajiri, Y. Model-Based Analysis of a Highly Efficient CO_2 Separation Process Using Flexible Metal–Organic Frameworks with Isotherm Hysteresis. *ACS Sustain. Chem. Eng.* **2022**, *10*, 14935–14947.

[12] Constant, N.; Liske, G.; Ravuru, S. S.; Puliyanda, A.; Pugnet, V.; Orsikowsky Sanchez, A.; Chavan, S. R.; Llewellyn, P.; Sawada, J. A.; Rajendran, A. Binary CO_2/H_2O Adsorption on CO_2 Capture Metal–Organic Frameworks CALF-20, Al-Fumarate and CAU-10-H Using Microscale Dynamic Column Breakthrough. *Ind. Eng. Chem. Res.* **2025**, *64*, 1712–1729.
